# Polymethoxy-1-Alkenes Screening of Chlorella and Spirulina Food Supplements Coupled with In Vivo Toxicity Studies

**DOI:** 10.3390/toxins12020111

**Published:** 2020-02-10

**Authors:** Eliana Henao, Patrick J. Murphy, Halina Falfushynska, Oksana Horyn, Daniel M. Evans, Piotr Klimaszyk, Piotr Rzymski

**Affiliations:** 1 Department of Biology, Universidad del Valle, 100-00 Cali, Colombia; eliana.henao@correounivalle.edu.co; 2 School of Natural Sciences, Alun Roberts Building (Chemistry), Bangor University, LL57 2UW Bangor, Wales; chs008@bangor.ac.uk; 3 Department of Orthopedagogy and Physical Therapy, Ternopil V. Hnatiuk National Pedagogical University, 46027 Ternopil, Ukraine; horynoi@tnpu.edu.ua; 4 Department of Water Protection, Adam Mickiewicz University, 61-614 Poznan, Poland; pklim@amu.edu.pl; 5 Department of Environmental Medicine, Poznan University of Medical Sciences, 60-806 Poznan, Poland

**Keywords:** cyanotoxin, polymethoxy-1-alkenes, food supplements, *Arthrospira*, Spirulina, *Chlorella*, human health risk assessment, zebrafish teratogenicity, oxidative stress, genotoxicity, neurotoxicity

## Abstract

Selected species of cyanobacteria and green algae have been reported to produce lipophilic polymethoxy-1-alkenes (PMAs) which were shown to exhibit in vivo teratogenicity. Considering that information on PMAs in *Arthospira* sp. (known commercially as Spirulina) and *Chlorella* sp. cultivated for food supplement production was essentially lacking, the present study screened Chlorella (*n* = 10) and Spirulina (*n* = 13) food supplements registered in the European Union. Mass spectrometry analysis of column fractionated extracts was performed. None of the four variants previously reported in some cyanobacteria and green algae, nor any potentially related structures were detected in the studied samples. Since the isolated lipophilic fractions contained various compounds, they were further screened for in vivo teratogenicity in *Danio rerio* embryo, and for the potential to induce oxidative stress and genotoxicity in the liver and neurotoxicity in the brain of adult zebrafish. None of the tested food supplements had detectable levels of PMAs or any potentially related structures. No teratogenicity was revealed except for spinal curvature induced by fractions obtained from two Chlorella products. Selected fractions revealed cytotoxicity as indicated by an increased level of reactive oxygen species, catalase activity, lipid peroxidation and increased frequency of DNA strand breaks in hepatic tissue. The majority (60%) of Chlorella fractions induced an increase in cholinesterase activity in zebrafish brain homogenate while exposure to 61.5% of Spirulina fractions was associated with its decrease. The present study confirms that Chlorella and Spirulina food supplements are free of teratogenic PMAs, although the observed in vivo toxicities raise questions regarding the quality of selected products.

## 1. Introduction

There is increasing interest in microalgal food supplements and other microalgae-based foodstuffs. The majority of these are based on *Chlorella* sp. and *Arthrospira* sp. (sold under the commercial name of “spirulina”), with the greatest production in Asia, particularly in China [1]. As evidenced, these products can be a source of proteins, vitamins and selected macro- and micro-elements, particularly iron [2,3,4]. A number of clinical trials on the therapeutic value of microalgal food supplements have been performed revealing their antihypertensive, antilipidemic, hypoglycemic and immunomodulatory effects [5,6,7].

Generally, the consumption of food supplements based on *Chlorella* or *Arthrospira* biomass has not been associated with health risks, and both are considered safe and are approved by food regulatory bodies such as the European Food Safety Agency (EFSA) or the Food and Drug Administration (FDA) [1]. However, over the years there have been some reports of an increased content of toxic metals, metalloids and cyanotoxins in selected commercial formulas which are attributed to the poor quality of production process (e.g., contaminated culture media, chemical methods of biomass harvesting, co-occurrence of toxigenic cyanobacteria species) [4,8,9,10]. The observed adverse effects mostly included mild gastrointestinal events such as diarrhea, nausea, abdominal cramps or vomiting [11,12].

**Figure 1:**
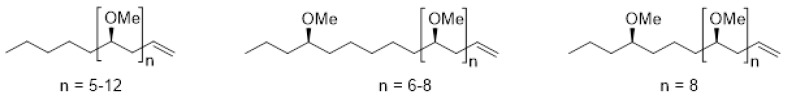
The four polymethoxy-1-alkenes identified so far in selected cyanobacteria and green algae.

Species belonging to the *Arthrospira* and *Chlorella* genera are not known to synthesize any toxic compounds that would cause human health risks. However, to our surprise and to the best of our knowledge, the presence of polymethoxy-1-alkenes (PMAs) in these supplements has never been subjected to study. PMAs are lipophilic compounds initially identified in cyanobacterium *Tolypothrix conglutinate*, and later shown to be present in various other freshwater microalgae, including cyanobacteria (*Aphanizomenon ovalisporum*, *Raphidiopsis raciborskii*, *Anabaena* sp., *Nostoc* sp., *Microcystis* sp., *Pseudanabaena* sp., *Scytonema burmanicum*, *S*. *ocellatum*, and *S*. *mirabilum*) and green algae (*Pediastrum* sp. and *Scenedesmus* sp.) [13,14,15,16,17,18]. As shown recently, the presence of PMAs may be strain-dependent or geographically diversified as no evidence was found for *A. gracile* or *R. raciborskii* isolated from lakes in Central Europe [19]. Overall, four PMAs varying in chain length have been identified to date (Figure 1). These compounds were also shown to reveal teratogenicity in a *Danio rerio* embryo experimental model [17,18]. However, the in vivo rodent models did not reveal any adverse effect of Chlorella or Spirulina supplementation on pregnancy outcome [20,21]; the use of the latter in pregnant women was shown by one study to correlate with oligohydramnios [22,23,24]. All in all, this highlights that exploring the presence of PMAs in commercially available microalgal supplements for human consumption is urgently needed. This is particularly important given the fact that these products are reported to be used by pregnant women [22], and some authors suggest that they may be beneficial during pregnancy and lactation as well as early childhood development due to their nutritional value [25,26].

In our previous research, we have successfully utilized nuclear magnetic resonance and mass spectrometry to screen PMA content in cyanobacteria *A. gracile* and *R. raciborskii* isolated from freshwater lakes in Poland. The mass spectrometry is however a method of choice as it is a much more sensitive and was utilized by more recent investigations [17,18,19]. The aim of this study was to investigate the PMA content in commercial Chlorella and Spirulina food supplements originating from cultivations in North America, Asia and Europe as well as to evaluate the toxicity of isolated lipophilic fractions using a zebrafish *Danio rerio* experimental model. The toxicological screening was performed because, beside PMAs, microalgae can produce a variety of different lipophilic toxic compounds, including teratogenic retinoic acids and carotenoid glycosides [27,28]. A set of assays was employed to evaluate whether the isolated fractions of food supplements can cause teratogenicity in zebrafish embryos as well as induce oxidative stress and genotoxicity in the liver, genotoxicity in peripheral red blood cells and neurotoxicity in the brain of adult zebrafish.

## 2. Results

### 2.1. Identification of Polymethoxy-1-Alkenes in Food Supplements

Ten commercially available Chlorella and thirteen Spirulina food supplements (Table 1) were screened for the content of PMAs using mass spectrometry following extraction of samples in CHCl_3_, drying under high vacuum and fractionation using silica gel chromatography eluting with a stepwise gradient of ethyl acetate in petrol (10%, 20%, 40% and 100%). Since no analytical standards were available and no database for PMAs exists, a comparison with literature reporting PMA detection using mass spectrometry was performed [17,18] in all of these fractions. The analysis was targeted towards the identification of known PMAs and also the presence of potential homologous compounds which contain extra [-CH=CH-]_n_, [-CH_2_CH(OMe)-]_n_ or [-CH_2_CH_2_-]_n_ fragments. None of these molecules were apparent in any studied Spirulina and Chlorella food supplement, and analysis of the major peaks did not correlate with these or any potentially related structures.

### 2.2. Toxicological Screening

An extracted lipophilic fraction (100% ethyl acetate) of each Chlorella (C1–C10) and Spirulina (S1–S13) food supplement was used to test teratogenicity in zebrafish embryo, oxidative stress and genotoxicity in the liver of adult specimens, genotoxicity in peripheral red blood cells and neurotoxicity in the brain.

#### 2.2.1. Evaluation of Teratogenicity of Lipophilic Fractions in Zebrafish Embryo

All control zebrafish embryos (both in Tris-buffer and Tris-buffer + 0.19% ethanol) met the acceptance criteria of >90% survival rate and normal development during the 120 h of the experiment. The exposure to lipophilic fractions of Chlorella and Spirulina food supplements did not produce any observable effect on survival and fingerprint endpoints, with two exceptions: C1 and C9 in which the scoliosis was induced (Table 2; Table 3). This effect was, however, recorded only after 96 h of exposure.

#### 2.2.2. Oxidative Stress in Liver of Adult Zebrafish

**Figure 2:**
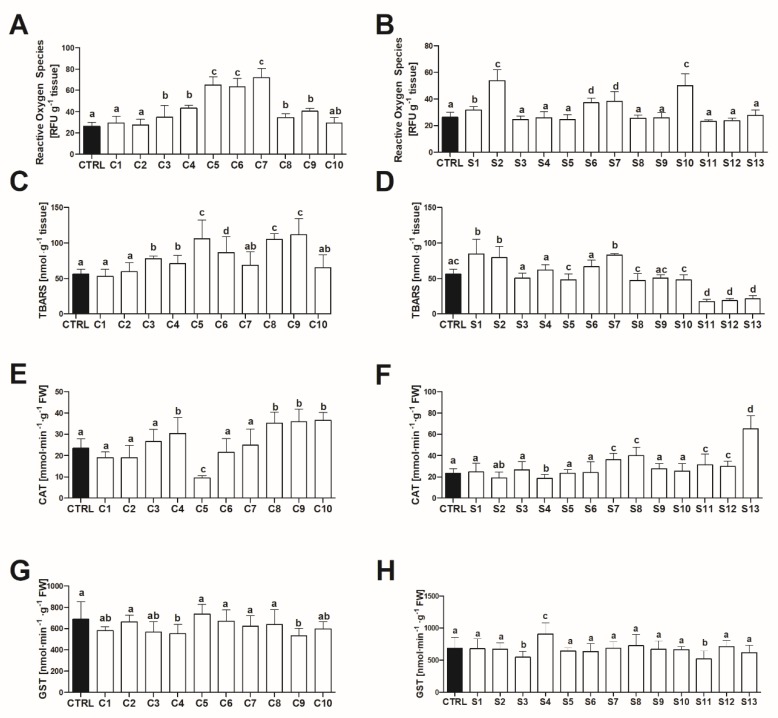
The effect (mean ± SD) of the lipophilic fraction (100% ethyl acetate) isolated from Chlorella (**A**,**C**,**E**,**G**) and Spirulina (**B**,**D**,**F**,**H**) food supplements on reactive oxygen species, lipid peroxidation (TBARS), catalase (CAT) and glutathione S-transferase (GST) activity in hepatic tissue of zebrafish (*n* = 6). Identical superscripts denote no significant differences between treatments (Dunn’s test after Kruskal–Wallis ANOVA, *p >* 0.05). CTRL–control.

The liver tissue collected from adult *D. rerio* revealed increased levels of reactive oxygen species (ROS) and lipid peroxidation (as measured by the content of thiobarbituric acid reactive substances; TBARS) following the exposure of fish to 7/10 and 6/10 fractions of Chlorella supplements, respectively. In comparison, ROS and TBARS were elevated after exposure to 5/13 and 3/13 fractions of Spirulina supplements (Figure 2). Moreover, the exposure to S11-S13 fractions was associated with a decrease in TBARS levels as compared to control. ROS and TBARS were in a positive correlation (*r* = 0.44, *p* < 0.001). Catalase (CAT) activity in hepatic tissue varied from oppression following exposure to C5 and S4 fractions, to two-fold elevation after exposure to the S13 fraction (Figure 2). The majority of fractions had no effect on GST activity, except for C4, C9, S3 and S11 when its decrease was observed, and for S4 when an increase was recorded. CAT activity revealed a negative correlation with levels of ROS and TBARS (*r* = −0.22, *p =* 0.007 and *r* = −0.30, *p =* 0.001 correspondingly), whereas no significant correlation for glutathione S-transferase (GST) activity and ROS/TBARS was found (*p* > 0.05).

#### 2.2.3. Geno and Neurotoxicity Signs in Adult Zebrafish

**Figure 3:**
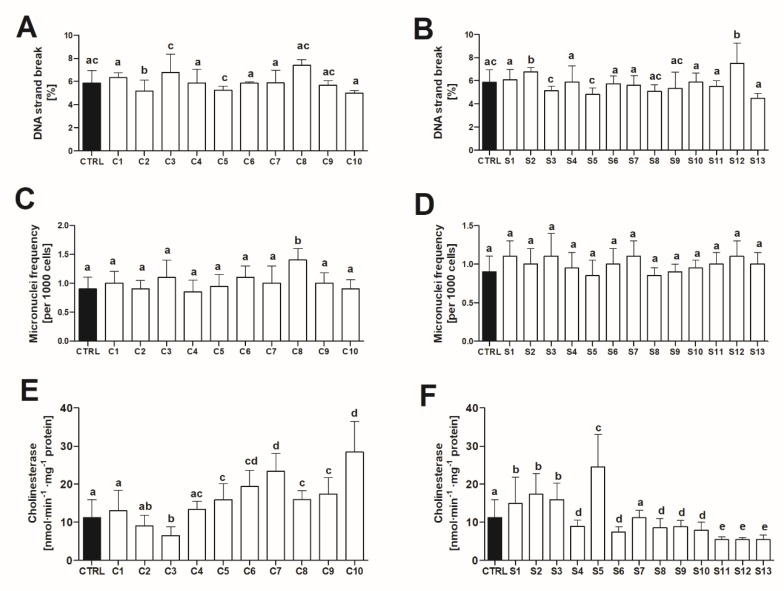
The effect (mean ± SD) of the lipophilic fraction (100% ethyl acetate) isolated from Chlorella (**A**,**C**,**E**) and Spirulina (**B**,**D**,**E**) food supplements on DNA strand breaks in hepatic tissue, micronuclei frequency in peripheral red blood cells and cholinesterase activity in the brain of zebrafish (*n* = 6). Identical superscripts denote no significant differences between treatments (Dunn’s test after Kruskal–Wallis ANOVA, *p* > 0.05). CTRL–control.

Most of tested fractions did not induce DNA strand breaks (DNAsb) in zebrafish liver. Exposure to any Spirulina sample was associated with an increased frequency of micronucleated peripheral red blood cells (Figure 3). Moreover, most of the Chlorella and Spirulina fractions tested induced changes in cholinesterase activity in fish brain with both, a significant decrease (C3, S4, S6, S8–S13) and increase (C5–C10, S1–S3, S5) noted (Figure 3).

## 3. Discussion

As postulated, ensuring the safety of food supplements based on natural products requires the implementation of analytical screening and the application of toxicity testing [29,30]. In the United States and the European Union, the safety of these products remains the responsibility of the manufacturer, while safety assurance is mostly limited to post-marketing surveillance for adverse effects [31]. Regulations have, however, been developed to lessen the possibility of food supplements being contaminated with toxic metals, herbicides or pathogenic microorganisms [31]. Nonetheless not all compounds of potential concern can be subject to random screening. Microalgae are known to produce a wide array of bioactive metabolites, while the precise structures of selected ones remain yet to be elucidated [32,33,34,35].

Surprisingly, the content of PMAs, which were found for the first time in cyanobacteria in 1979 [13] and were later proven to be teratogenic [17], has never been investigated for Chlorella or Spirulina food supplements. The broadest screening of these compounds in green algae and cyanobacteria, encompassing a total of 98 strains, was performed by Jaja-Chimedza et al. [18]. However, this study investigated only one isolate of *Arthrospira* sp. [18] while the production of bioactive metabolites in microalgae, including PMAs in cyanobacteria, has been shown to be geographically diversified and often strain-dependent [19,36]. Importantly, such strain-dependency in the production of various compounds, which are present only in small quantities in microalgal biomass, was also documented for strains belonging to the *Arthrospira* genus [37]. Moreover, one should note that even if PMAs were not produced by any strain of *Chlorella* and *Arthrospira* cultivated for human consumption, the presence of these compounds in food supplements may potentially originate from culture contamination with other microalgal species. For example, the occurrence of hepatotoxic microcystins in selected food products based on *Chlorella* and *Arthrospira* biomass has been linked to the co-occurrence of toxigenic cyanobacteria such as *Microcystis* [38,39,40,41,42]. The present study rules out the presence of teratogenic PMAs or related structures in both Spirulina and Chlorella food supplements and can confirm consumer safety in this respect.

Nevertheless, one should note that PMAs are not the only teratogenic compounds which could potentially be present in microalgal cells. For example, selected cyanobacteria and green-algae strains have been found to produce retinoic acids and carotenoid glycosides which triggered a diverse set of teratogenic effects in the *Danio rerio* experimental model [27,28,43]. No *Arthrospira* strains were investigated for the presence of these compounds, while in the case of *Chlorella* genus, retinoid-like activities were only ruled out for *Chlorella kessleri* [27]. Therefore, in the present study, we also assessed teratogenicity using the well-established zebrafish embryo assay [44]. As demonstrated, no significant or marked effects on embryo/larvae development were found for the lipophilic fractions obtained from food supplements. The only exception was the spinal curvature induced by two Chlorella samples. The causative agent remains unknown. If one considers that the majority of food supplements from this group did not produce such an effect, it can be postulated that it is likely due to the result of the low quality and potential contamination of selected products. Some Chlorella-based supplements have been shown to contain various polycyclic aromatic hydrocarbons (PAHs) [45,46], and these compounds are known to induce various teratogenic effects [47]. The content of PAHs in microalgal supplements would, however, require a comprehensive screening.

To further explore potential toxicities of lipophilic fractions obtained from the tested food supplements, their potential to induce oxidative stress, genotoxicity and neurotoxicity was also assessed in vivo using adult zebrafish. As demonstrated, selected samples increased the level of ROS, CAT activity, lipid peroxidation (as indicated by the TBARS assay) and increased frequency of DNA strand breaks in hepatic tissue. This indicates that these fractions possessed cytotoxic potency. The cytotoxicity of extracts of Chlorella and Spirulina food supplements were previously demonstrated in vitro in A549 cells, although none of the tested products contained detectable levels of cyanotoxins, such as microcystins, nodularins, saxitoxins, anatoxin-a and cylindrospermopsin [48]. Case reports of hepatotoxicity associated with the use of food supplements based on Chlorella and Spirulina were also reported [11,49,50]. All in all, this highlights that the safety of selected microalgal products is questionable. However, one should bear in mind that the fractions of three Spirulina supplements tested in the present study decreased the level of lipid peroxidation as compared to the control which is in line with the hepatoprotective properties of these products reported previously in vitro, in vivo and in clinical trials [51,52].

As demonstrated in the present study, 60% and 31% of fractions of Chlorella and Spirulina, respectively, induced an increase in cholinesterase activity in zebrafish brain homogenate. This indicates that these fractions could contain compounds causing a potential loss of cholinergic homeostasis leading to acetylcholine degradation and downregulation of acetylcholine receptors [53]. In turn, the exposure to 61.5% of Spirulina fractions (and one fraction of Chlorella) was associated with a decrease in cholinesterase activity, which may be associated with increased levels of acetylcholine and overstimulation of cholinergic stimulation [54]. Both an increase and decrease in cholinesterase activity can be associated with adverse effects, although their potential occurrence would require further assessment. There are no known reports of neurotoxicity following the use of Chlorella and Spirulina. However, the findings of the present study suggest that both groups of food supplements may contain lipophilic compounds possessing potential neuromodulatory activities.

## 4. Conclusions

The present study highlights that commercially available microalgal food supplements based on biomass of Spirulina and Chlorella are free of PMAs, while the lipophilic fractions obtained from most of these products do not reveal teratogenicity in a zebrafish model. However, some fractions revealed cytotoxicity and neurotoxicity and affected cholinesterase activity. This indicates that the quality and safety of selected microalgal products may be questionable.

## 5. Materials and Methods

### 5.1. Food Supplements

A total of 10 Chlorella-based and 13 Spirulina-based food supplements were randomly purchased from Polish online stores. The inclusion criteria were official registration as a food supplement, tablet or powder form and country of origin declared on the label. The general characteristics of the studied products and the region of origin are summarized in Table 1.

### 5.2. Sample Extraction

The whole batch of each supplement was ground and thoroughly mixed. The powdered samples (10 g) were suspended in CHCl_3_ (50 mL) and stirred vigorously for 16 h then filtered using a sintered filter apparatus. This procedure was repeated a total of three times and the combined extracts dried (MsSO_4_), filtered, the solvent removed under reduced pressure and the residue obtained dried under high vacuum to a constant weight (typically between 5% to 15% by mass of extract was obtained; see Supplementary Material for full details). The material obtained was fractioned using silica gel chromatography eluting with a stepwise gradient of 200 mL each of: 10%, 20%, 40% and 100% ethyl acetate in petrol. Each fraction was collected and evaporated to dryness using a rotary evaporator (Buchi R124 Rotavapor, Flawil, Switzerland) and the ethyl acetate fraction was typically between 0.5% to 3.0% by mass of the original supplement (see Supplementary Material for full details).

### 5.3. Mass Spectrometry

To identify PMAs in the obtained fractions a mass spectrometry analysis was performed with an LTQ Orbitrap XL Mass Spectrometer (Thermo Fisher, Altrincham, UK). Initial characterization of samples was made by atmospheric pressure chemical ionization (APCI) mass spectrometry via an Atmospheric Solids Analysis Probe (ASAP) on a Waters Xevo G2-S instrument. A small amount of solid sample was transferred to the tip of a glass capillary, which was then placed within the ASAP source and inserted into the instrument. The vaporizer temperature was increased from 50 °C to a temperature at which ions were observed and acquired; the discharge current was 4 μA. Data was processed using vendor MassLynx software. Since no analytical standards were available and no database for these compounds exists, a comparison with literature was performed [17,18].

### 5.4. Toxicological Assays

All zebrafish exposures were conducted at 18 ± 1 °C. Adult 6-month old zebrafish were obtained from a commercial supplier (Ukraine). The fish underwent an initial acclimation for 7 days and then were randomly distributed to the control, Spirulina (S) and Chlorella (C) exposure groups, with 3 replicate tanks per group, and six specimens per replicate. During acclimation and experimental treatment, *D. rerio* were fed ad libitum with a commercial food. Water was changed every two days. For Spirulina and Chlorella exposures, a static renewal design was used with algae extract addition during each water change. The exposure treatment was continued for 14 days. Clear solution of lipophilic fractions (100%) of Spirulina and Chlorella in 2 mL 96% ethanol was added to the experimental tanks to a nominal concentration of 200 µL L^−1^ by means of dilution of stock solution of lipophilic fractions into 5000 times. Ethical manifestations of Institutional Animal Care and Use Committee protocols (The Institutional Animal Care and Use Committee of the Ternopil V. Hnatiuk National Pedagogical University approved experimental protocols and sampling procedures (approval no. 3/2019, approval date: 3 September 2019). National and international guidelines for the care and use of fisheries were followed. The tissues were sampled according to the Guide for the Care and Use of Laboratory Animals.

After exposure, the zebrafish were anesthetized by tricaine. Samples of hepatic tissue and peripheral blood from six specimens in each group were prepared individually and kept at −20 °C (for less than three days) until analyses. Hepatic and brain tissue was homogenized (1:10 w/v) at 4 °C in 0.1 M pH 7.4 phosphate buffer containing 100 mM KCl, 1 mM EDTA and 0.1 mM phenylmethylsulfonylfluoride. The homogenate was centrifuged at 6000× *g* for 10 min at 4 ° C. Protein concentration in the supernatant was measured according to Lowry et al. (1951) with bovine serum albumin as a standard. The absorbance was measured with a UV/Vis spectrophotometer, and the fluorescence was measured on the f-max fluorescence microplate reader (Molecular Devices, San Jose, CA, USA).

#### 5.4.1. Embryotoxicity Testing in Danio Rerio

The embryo toxicity test was carried out using *D. rerio* embryos in accordance with the OECD [55]. In brief, adult specimens of wild-type zebrafish (older than 6 months) with high potential to lay fertilized eggs were chosen to spawn, and then, the standard method of breeding was used [55]. Egg production was from female and male spawning groups at a ratio of 1:2, correspondingly. Mating and spawning occurred within 30 min after turning on the lights in the morning. Egg laying was covered with a plastic mesh for protection against predation. Approx. 30 min after the light exposure, the egg traps were removed, and the eggs were collected. Following this, the eggs were rinsed in 0.0002% methylene blue (Sigma-Aldrich, St. Louis, MO, USA), diluted in medium and placed into Petri dishes. Within 2 h post-fertilization healthy fertilized eggs were selected for the subsequent embryo toxicity test. Zebrafish embryos (*n* = 6) were exposed in a 24-well plate filled with 2 mL Tris-buffer containing 0.4 µL of a lipophilic fraction (100%) of each supplement in 2 mL 96% ethanol at 26 °C with a 12:12-h light/dark cycle. The observed endpoints within 120 h exposure included mortality, egg coagulation and morphological changes, among which tail and chorda malformation, spinal curvature, otolith and eyes were recorded. The endpoint had to be reflected in ≥50% of all embryos and/or larvae to indicate the teratogenic effect.

#### 5.4.2. Oxidative Stress Assays

The rates of reactive oxygen species formation in the liver homogenates in 0.1 M phosphate buffer pH 7.4 were determined using dihydrorhodamine, which is converted by reactive oxygen species to the fluorescent compound rhodamine-123 [56]. The fluorescence signal was registered by an f-max plate-reader [excitation = 485 nm, emission = 538 nm], and the rates of reactive oxygen species formation were determined as the difference of the fluorescence at time 0 and after 20 min of incubation and expressed as relative fluorescence unite (RFU) per 1 g of liver tissue. Lipid peroxidation (LPO) was determined in the supernatant of the liver tissue homogenate (1:10 m/v) in 0.1 M phosphate buffer pH 7.4 using thiobarbituric acid protocol. The level of thiobarbituric acid-reactive substances (TBARS) was measured colorimetrically at 532 nm [57] and calculated using a molar extinction coefficient of 1.56 × 10^5^ M^−1^·cm^−1^. Catalase (CAT) activity was determined in the supernatant of liver tissue homogenate (1:10 w:v) in 0.1 M pH 7.4 phosphate buffer by following the decrease in hydrogen peroxide at 240 nm [58]. CAT activity was determined using the extinction coefficient, *ε* = 40 M^−1^·cm^−1^ and expressed as mmol × min^−1^ × g^−1^ tissue. Glutathione S-transferase (GST) activity was measured in the *D. rerio* liver tissue homogenate (1:10 m/v) using 1-chloro-2,4- dinitrobenzene (CDNB) reduction assay [59]. GST activity was determined as the CDNB absorbance difference over time using an extinction coefficient of 9.6 mM^−1^ cm^−1^ and expressed as nmol g^−1^ tissue.

#### 5.4.3. Genotoxicity Assays

To analyze the genotoxicity of lipophilic fractions isolated from Chlorella and Spirulina food supplements, levels of protein-free DNA strand breaks in zebrafish liver tissue homogenate were determined by the alkaline DNA precipitation assay [60] based on using Hoechst 33342 dye in the presence of 4 mM sodium cholate [61]. The fluorescence signal was detected by an *f*-max fluorescence plate-reader (excitation = 360 nm, emission = 450 nm). The amount of protein-free DNA strand breaks was referred to the concentration of the protein in the sample. The genotoxic effect of model exposures was assessed as the micronucleated erythrocytes in the peripheral blood of adult *D. rerio* using Giemsa stain protocol [62]. The frequency of micronuclei was expressed per 1000 cells.

#### 5.4.4. Neurotoxicity Assay

Cholinesterase (ChE, EC 3.1.1.7) activity was determined in the zebrafish brain as acetylthiocholine-cleaving ChE activity according to the colorimetric method of Ellman et al. [63]. ChE activity was calculated using a molar extinction coefficient of 13.6 × 10^3^ М^–1^·сm^–1^ and referred to the soluble protein content.

## Figures and Tables

**Table 1:** The general characteristics of studied Chlorella (*n* = 10) and Spirulina (*n* = 13) food supplements.

Sample Code	Species Declared on the Label	Country of Origin	Appearance
**Chlorella**			
C1	*Chlorella* sp.	China	Tablets
C2	*Chlorella vulgaris*	Taiwan	Tablets
C3	*Chlorella pyrenoidosa*	Japan	Tablets
C4	*Chlorella* sp.	India	Tablets
C5	*Chlorella* sp.	China	Powder
C6	*Chlorella* sp.	China	Powder
C7	*Chlorella pyrenoidosa*	China	Powder
C8	*Chlorella vulgaris*	China	Powder
C9	*Chlorella vulgaris*	Portugal	Powder
C10	*Chlorella vulgaris*	China	Tablets
**Spirulina**			
S1	*Spirulina* sp.	China	Powder
S2	*Spirulina* sp.	China	Powder
S3	*Spirulina* sp.	China	Powder
S4	*Spirulina platensis*	China	Powder
S5	*Spirulina* sp.	Taiwan	Powder
S6	*Spirulina pacifica*	USA	Tablets
S7	*Arthrospira platensis*	China	Powder
S8	*Spirulina platensis*	China	Tablets
S9	*Spirulina* sp.	China	Tablets
S10	*Spirulina maxima*	China	Tablets
S11	*Spirulina* sp.	China	Tablets
S12	*Spirulina* sp.	China	Tablets
S13	*Spirulina* sp.	India	Powder

**Table 2:** The effect of lipophilic fractions (100% ethyl acetate) isolated from Chlorella food supplements (C1-C10) on teratogenicity in zebrafish embryos (*n* = 6) exposed in a static manner for 120 h.

Developmental Endpoint	Sample										
	CTRL	C1	C2	C3	C4	C5	C6	C7	C8	C9	C10
Coagulated eggs	-	-	-	-	-	-	-	-	-	-	-
Head malformation	-	-	-	-	-	-	-	-	-	-	-
Eyes malformation	-	-	-	-	-	-	-	-	-	-	-
Chorda malformation	-	-	-	-	-	-	-	-	-	-	-
Tail malformation	-	-	-	-	-	-	-	-	-	-	-
Egg yolk malformation	-	-	-	-	-	-	-	-	-	-	-
Growth retardation	-	-	-	-	-	-	-	-	-	-	-
Spinal curvature	-	-	+	-	-	-	-	-	-	+	-

CTRL, control; “-“, normal; “+”, abnormal (reflected in ≥50% of all embryos and/or larvae).

**Table 3:** The effect of lipophilic fractions (100% ethyl acetate) isolated from Spirulina food supplements (S1–S13) on teratogenicity in zebrafish embryos (*n* = 6) exposed in a static manner for 120 h.

Developmental Endpoint	Sample													
	CTRL	S1	S2	S3	S4	S5	S6	S7	S8	S9	S10	S11	S12	S13
Coagulated eggs	-	-	-	-	-	-	-	-	-	-	-	-	-	-
Head malformation	-	-	-	-	-	-	-	-	-	-	-	-	-	-
Eyes malformation	-	-	-	-	-	-	-	-	-	-	-	-	-	-
Chorda malformation	-	-	-	-	-	-	-	-	-	-	-	-	-	-
Tail malformation	-	-	-	-	-	-	-	-	-	-	-	-	-	-
Egg yolk malformation	-	-	-	-	-	-	-	-	-	-	-	-	-	-
Growth retardation	-	-	-	-	-	-	-	-	-	-	-	-	-	-
Spinal curvature	-	-	-	-	-	-	-	-	-	-	-	-	-	-

CTRL–control; “-“, normal; “+”, abnormal (reflected in ≥50% of all embryos and/or larvae).

## Supplementary Materials

The following are available online at https://www.mdpi.com/2072-6651/12/2/111/s1.
