# Supplementary material for: Polymethoxy-1-Alkenes Screening of Chlorella and Spirulina Food Supplements Coupled with In Vivo Toxicity Studies
Source: Toxins (Basel). 2020 Feb 10;12(2):111. doi: 10.3390/toxins12020111 (PMC7077266; doi:10.3390/toxins12020111)

Mass Spectrometry Details

In-house analysis Thermo Fisher LCQ Deca.

ESI source:
Ionization mode: positive
Sheath gas flow rate: 35
Sweep gas flow rate: 0
Spray voltage: 4.5kV
Capillary temperature: 250°C
Capillary voltage: 3V
Tube lens offset: -30V

Injection control:
AGC settings:
FullMS Target: 5e7

Ion optics:
Multipole 1 Offset: -1.25V
Lens voltage: -16V
Multipole 2 Offset: -11V
Multipole RF Amplitude: 400V
Enhance Lens: -68V

NMSF Atmospheric Solids Analysis Probe (ASAP)

APCI on a Waters Xevo G2-S instrument.

ASAP analyses: Initial characterisation of samples was made by atmospheric pressure chemical ionisation (APCI) mass spectrometry via an atmospheric solids analysis probe (ASAP) on a Waters Xevo G2-S instrument. A small amount of solid sample was transferred to the tip of a glass capillary, which was then placed within the ASAP source and inserted into the instrument. The vaporizer temperature was increased from 50°C to a temperature at which ions were observed and acquired and the discharge current was 4 μA . Data was processed using vendor MassLynx software.

Please also see the header of each spectrum.

E:\dan_evans\RAW FILES\100%\CH1 08/16/18 13:27:31 CH1

CH1 #1-50 RT: 0.01-0.75 AV: 50 NL: 2.38E5
T: + p ESI ms [150.00-1000.00]

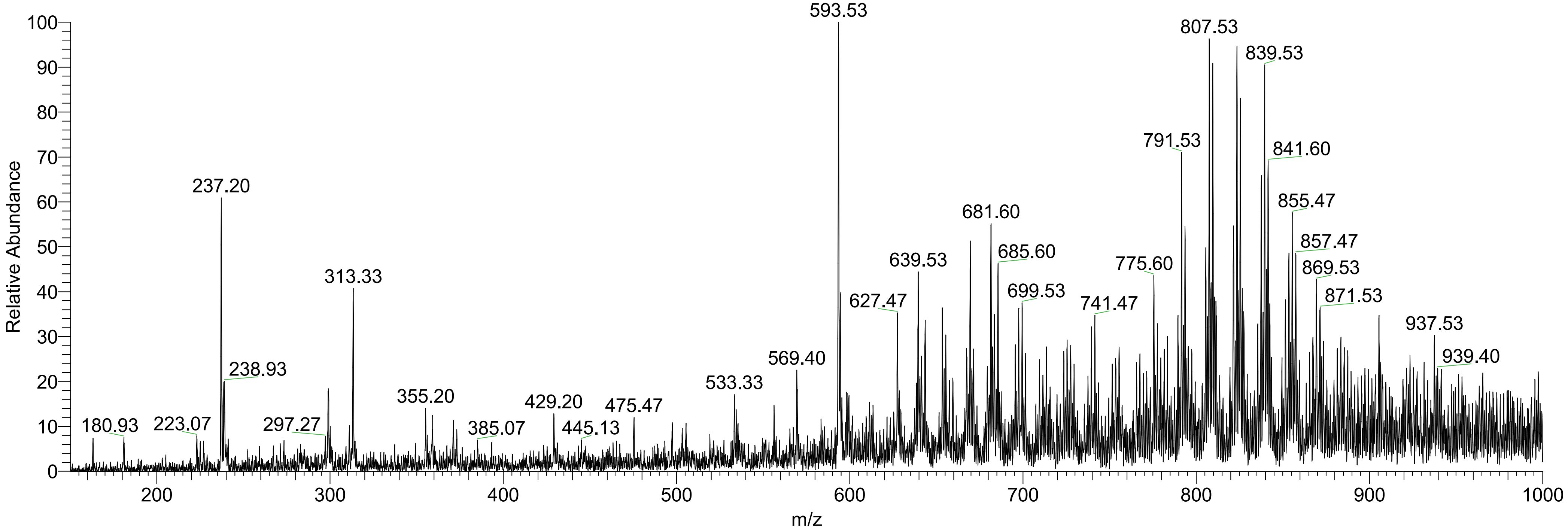

CH1 #1-50 RT: 0.01-0.75 AV: 50 NL: 2.38E5
T: + p ESI ms [150.00-1000.00]

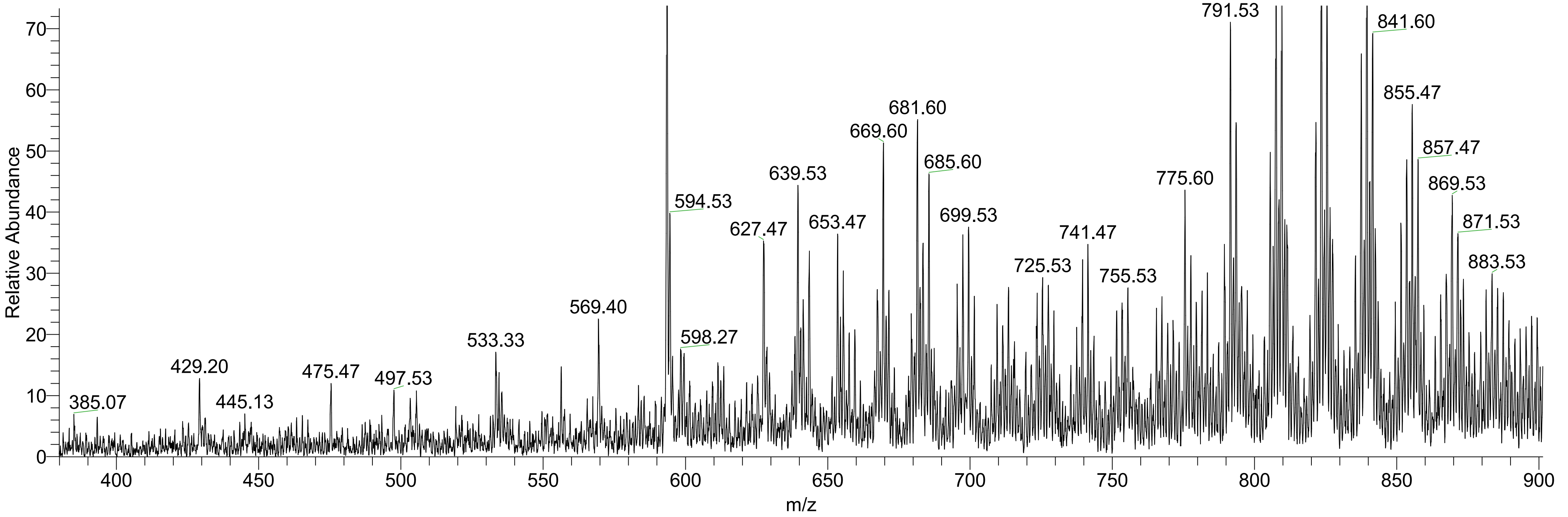

E:\dan_evans\RAW FILES\100%\CH2 08/16/18 13:41:43 CH2

CH2 #1-50 RT: 0.00-0.74 AV: 50 NL: 1.08E6
T: + p ESI ms [150.00-1000.00]

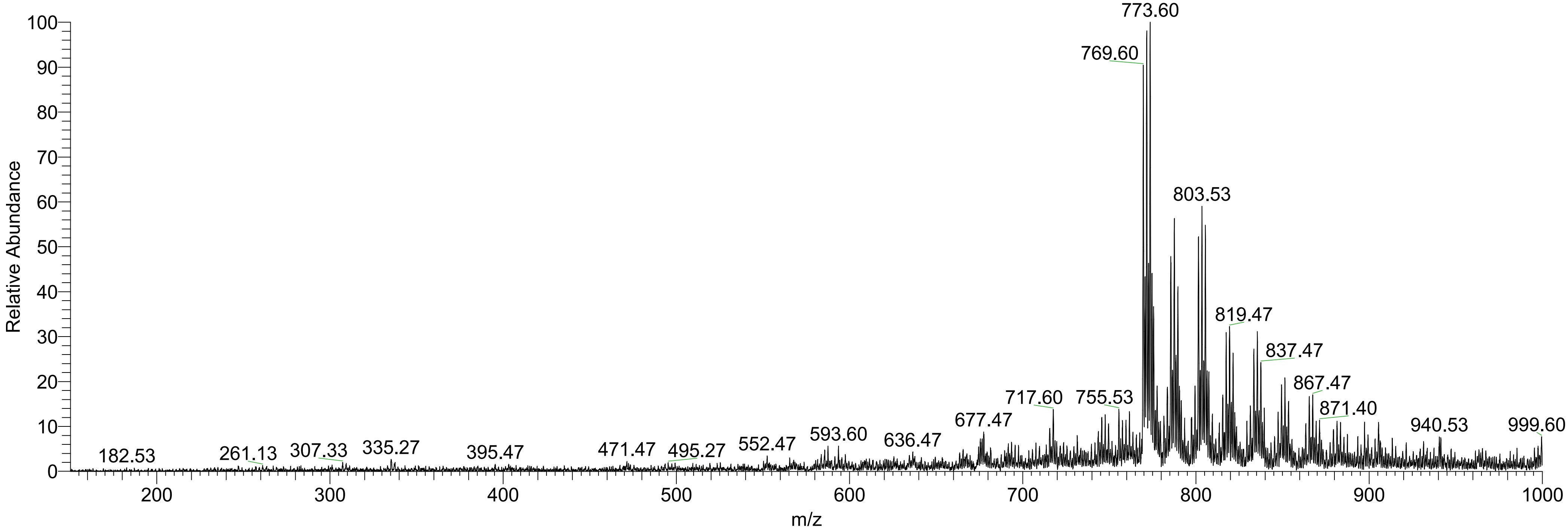

CH2 #1-50 RT: 0.00-0.74 AV: 50 NL: 1.08E6
T: + p ESI ms [150.00-1000.00]

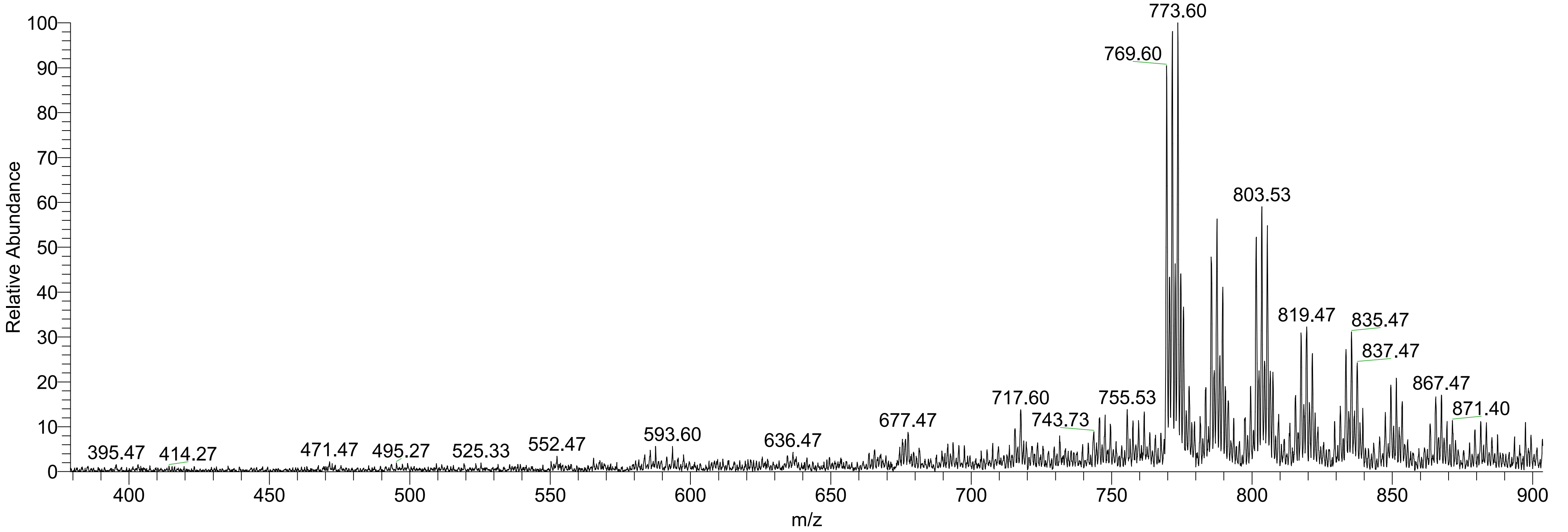

E:\dan_evans\RAW FILES\100%\CH3 08/16/18 13:48:27 CH3

CH3 #1-50 RT: 0.01-0.74 AV: 50 NL: 8.70E5
T: + p ESI ms [150.00-1000.00]

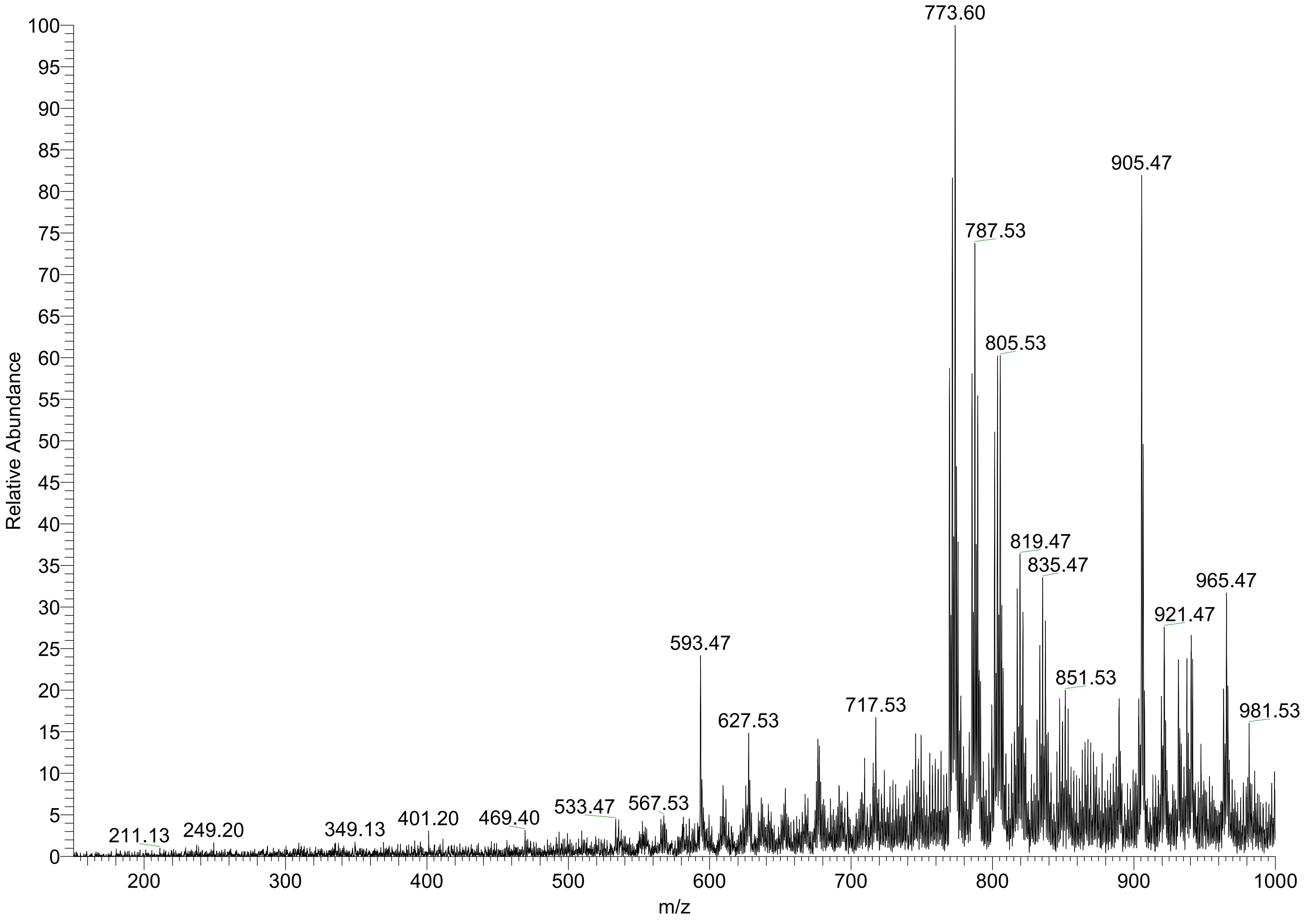

E:\dan_evans\RAW FILES\100%\CH3 08/16/18 13:48:27 CH3

CH3 #1-50 RT: 0.01-0.74 AV: 50 NL: 8.70E5
T: + p ESI ms [150.00-1000.00]

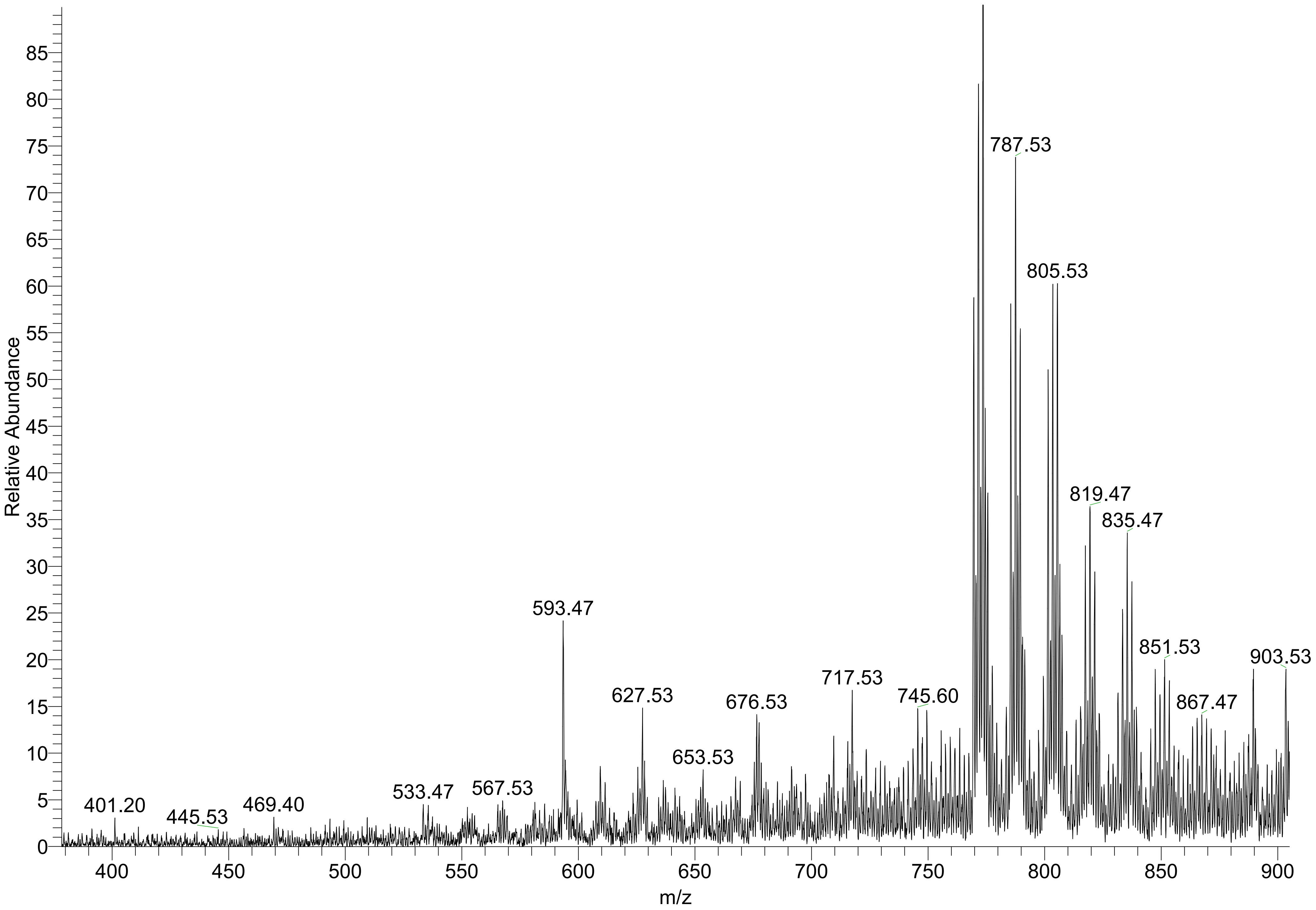

E:\dan_evans\RAW FILES\100%\CH4 08/16/18 13:55:06 CH4

CH4 #1-50 RT: 0.00-0.74 AV: 50 NL: 7.04E5
T: + p ESI ms [150.00-1000.00]

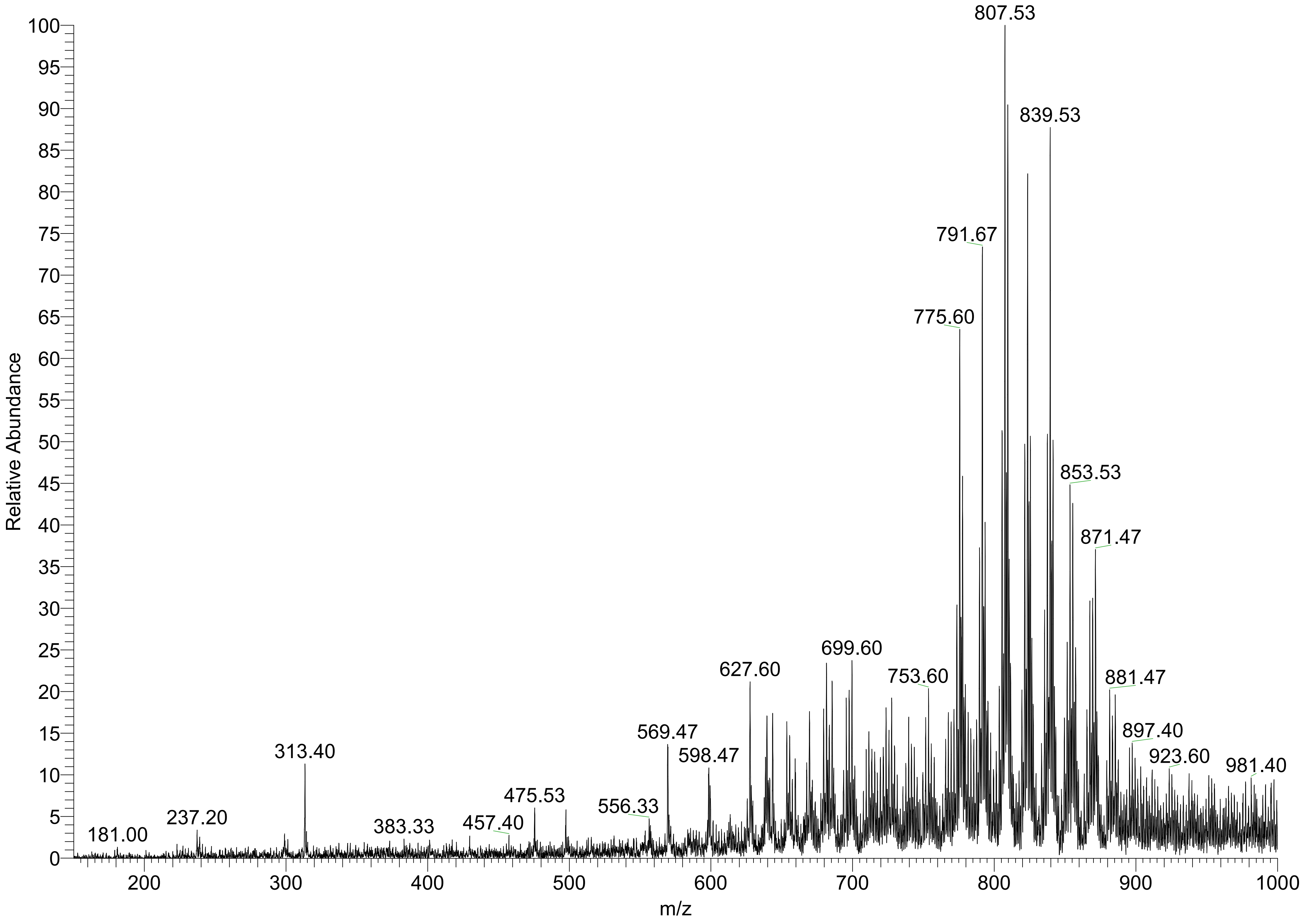

E:\dan_evans\RAW FILES\100%\CH4 08/16/18 13:55:06 CH4

CH4 #1-50 RT: 0.00-0.74 AV: 50 NL: 7.04E5
T: + p ESI ms [150.00-1000.00]

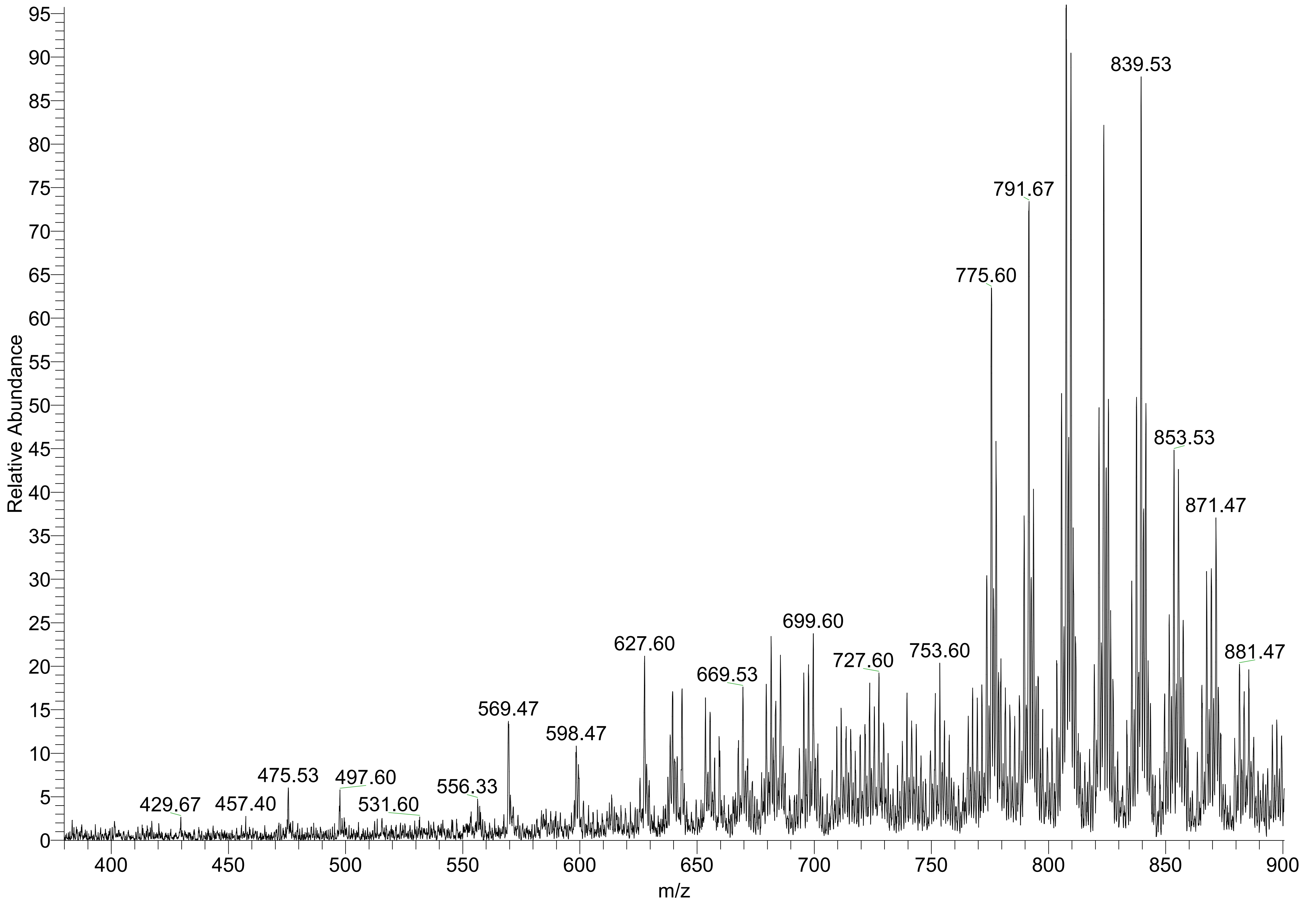

E:\dan_evans\RAW FILES\100%\CH5 08/16/18 14:00:03 CH5

CH5 #1-50 RT: 0.01-0.75 AV: 50 NL: 9.18E5
T: + p ESI ms [150.00-1000.00]

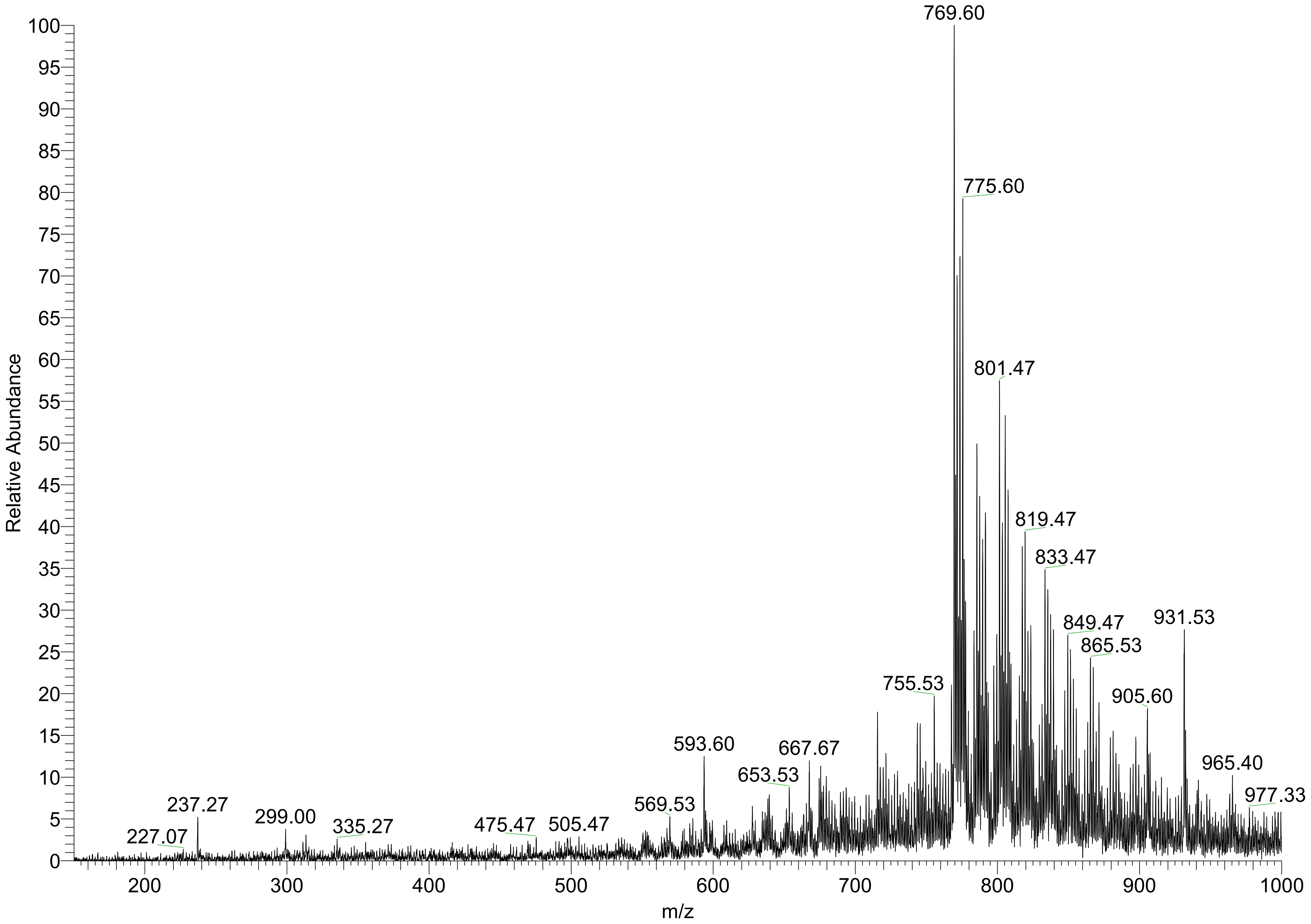

E:\dan_evans\RAW FILES\100%\CH5 08/16/18 14:00:03 CH5

CH5 #1-50 RT: 0.01-0.75 AV: 50 NL: 9.18E5
T: + p ESI ms [150.00-1000.00]

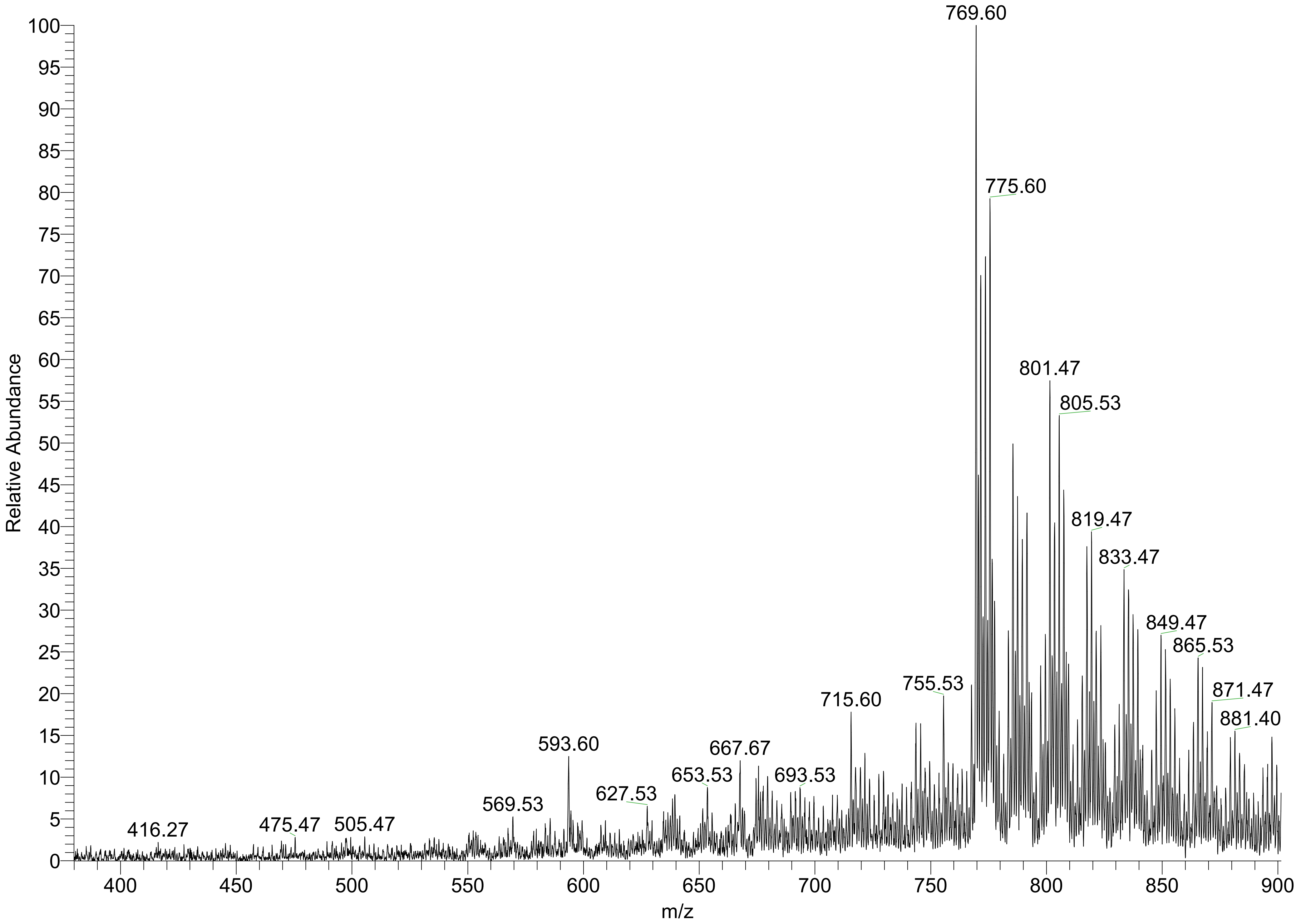

E:\dan_evans\RAW FILES\100%\CH6 08/16/18 14:05:17 CH6

CH6 #1-50 RT: 0.00-0.74 AV: 50 NL: 3.12E6
T: + p ESI ms [150.00-1000.00]

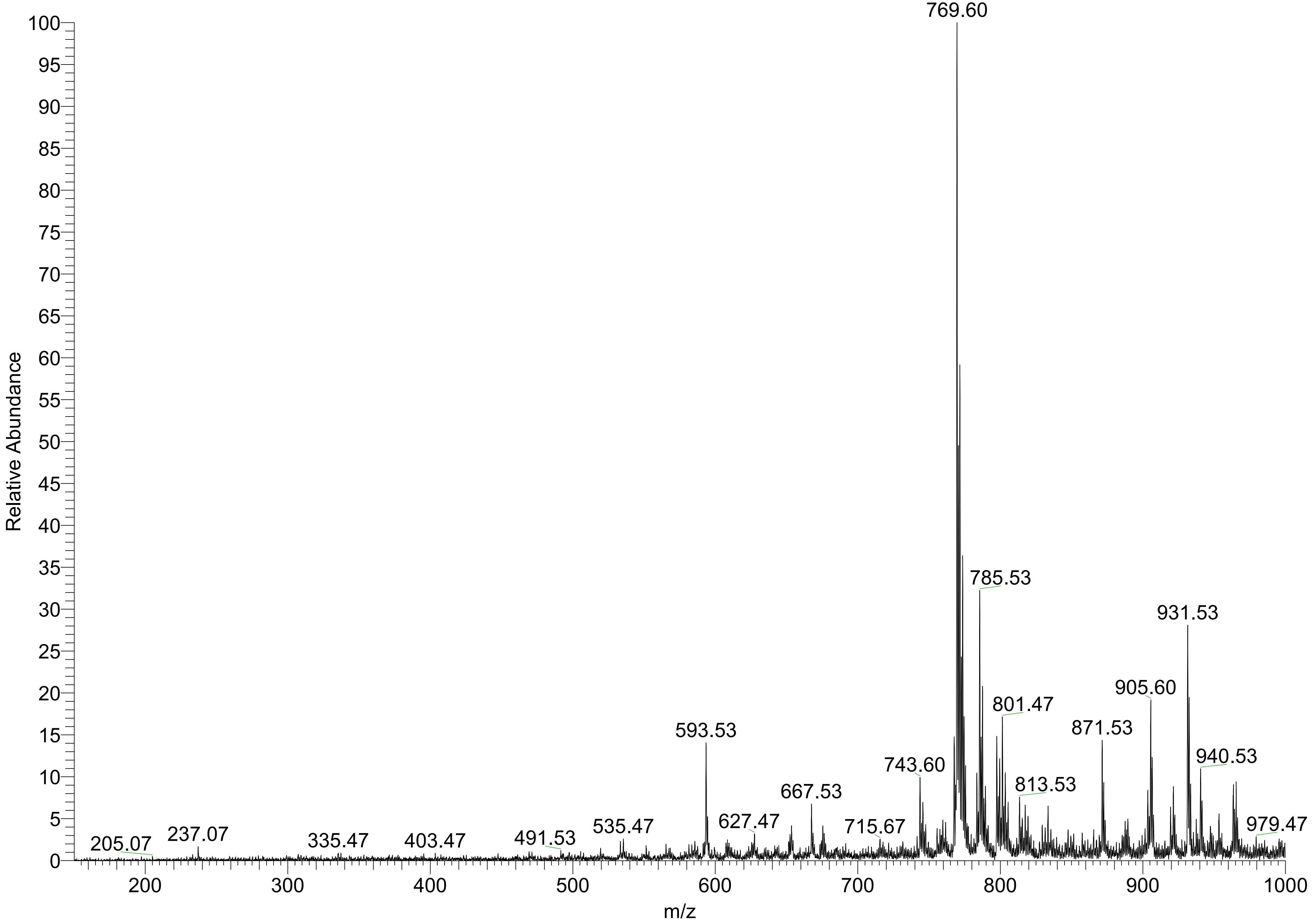

E:\dan_evans\RAW FILES\100%\CH6 08/16/18 14:05:17 CH6

CH6 #1-50 RT: 0.00-0.74 AV: 50 NL: 3.12E6
T: + p ESI ms [150.00-1000.00]

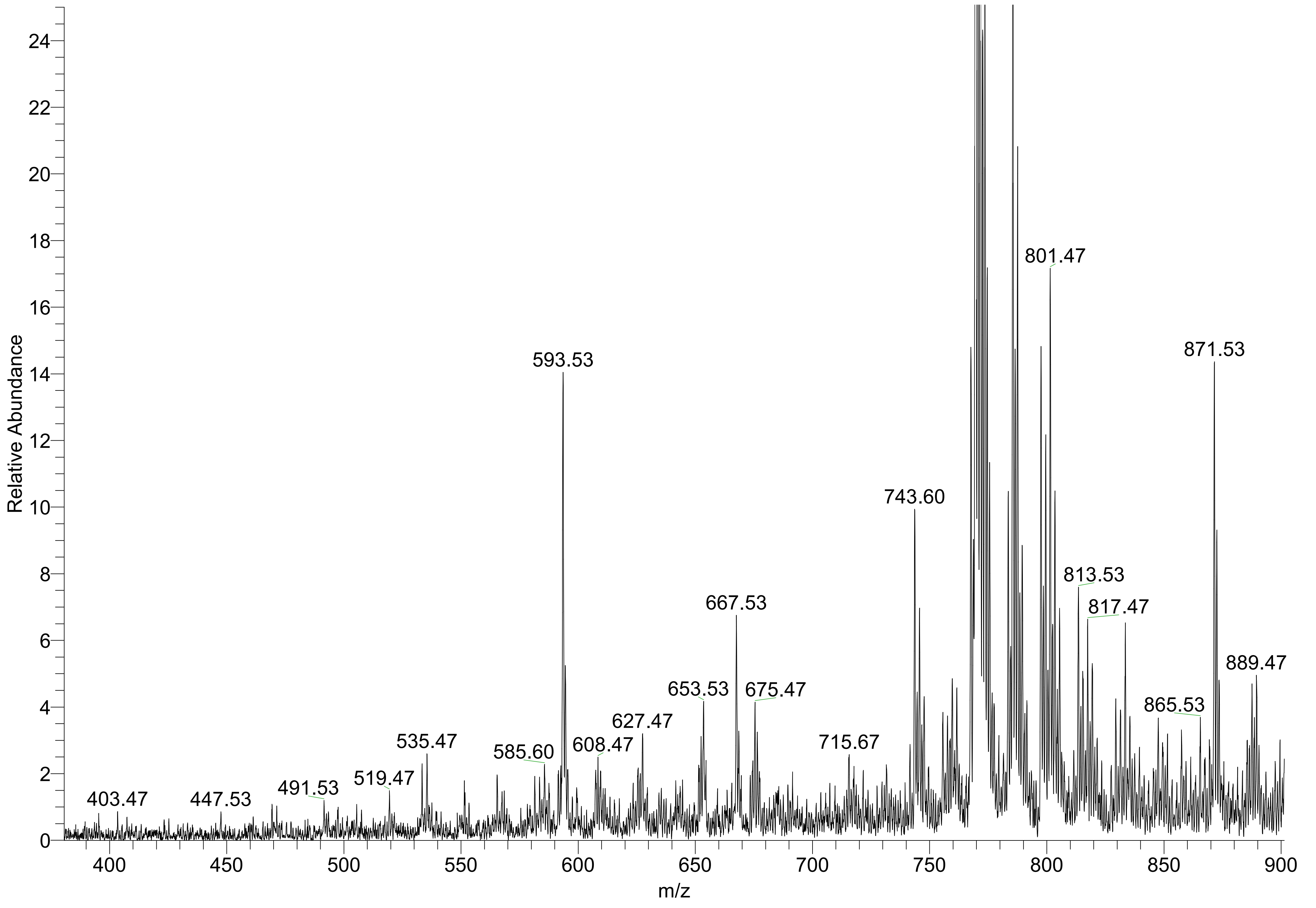

E:\dan_evans\RAW FILES\100%\CH7 08/16/18 14:10:17 CH7

CH7 #1-50 RT: 0.01-0.75 AV: 50 NL: 2.45E6
T: + p ESI ms [150.00-1000.00]

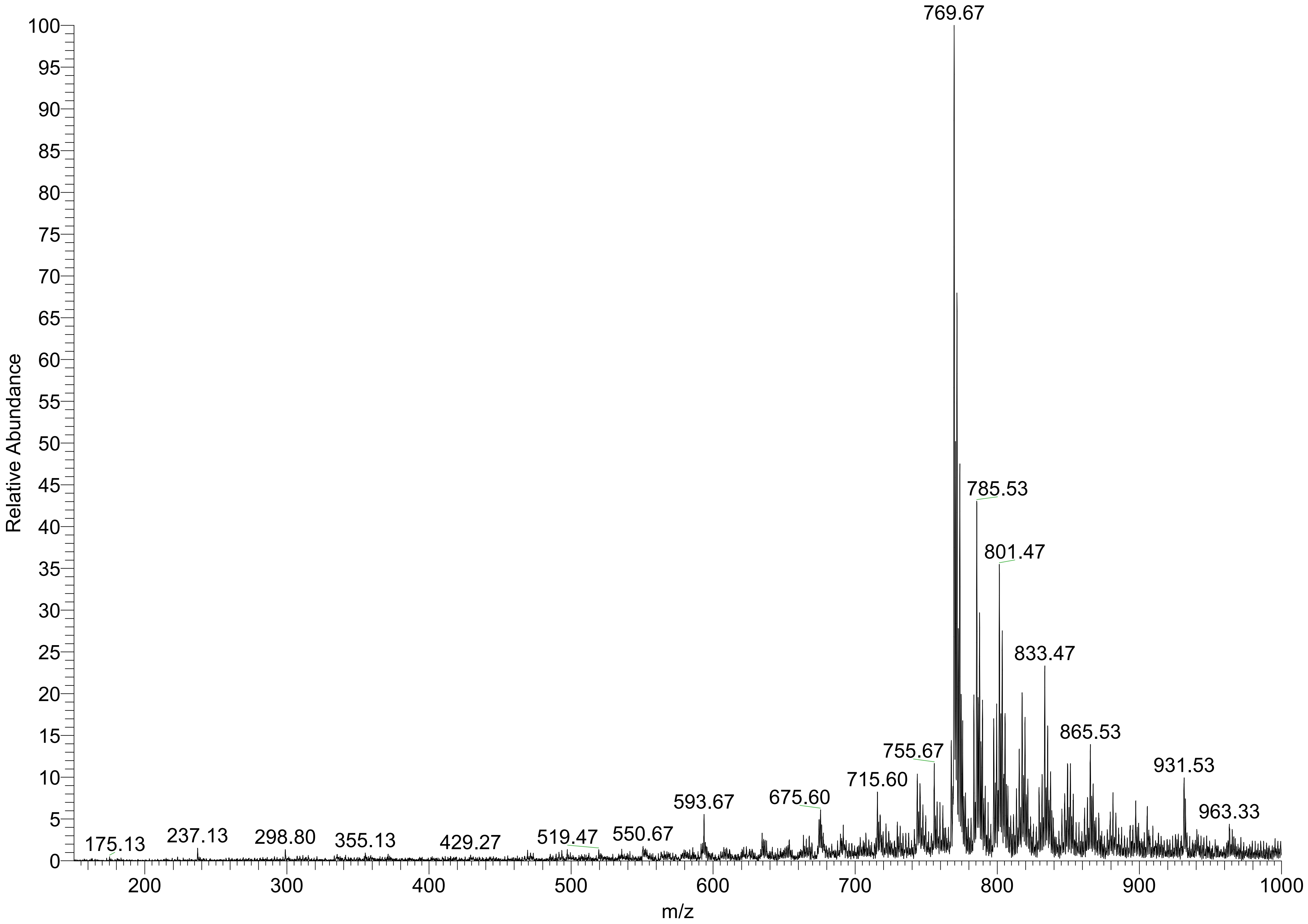

E:\dan_evans\RAW FILES\100%\CH7 08/16/18 14:10:17 CH7

CH7 #1-50 RT: 0.01-0.75 AV: 50 NL: 2.45E6
T: + p ESI ms [150.00-1000.00]

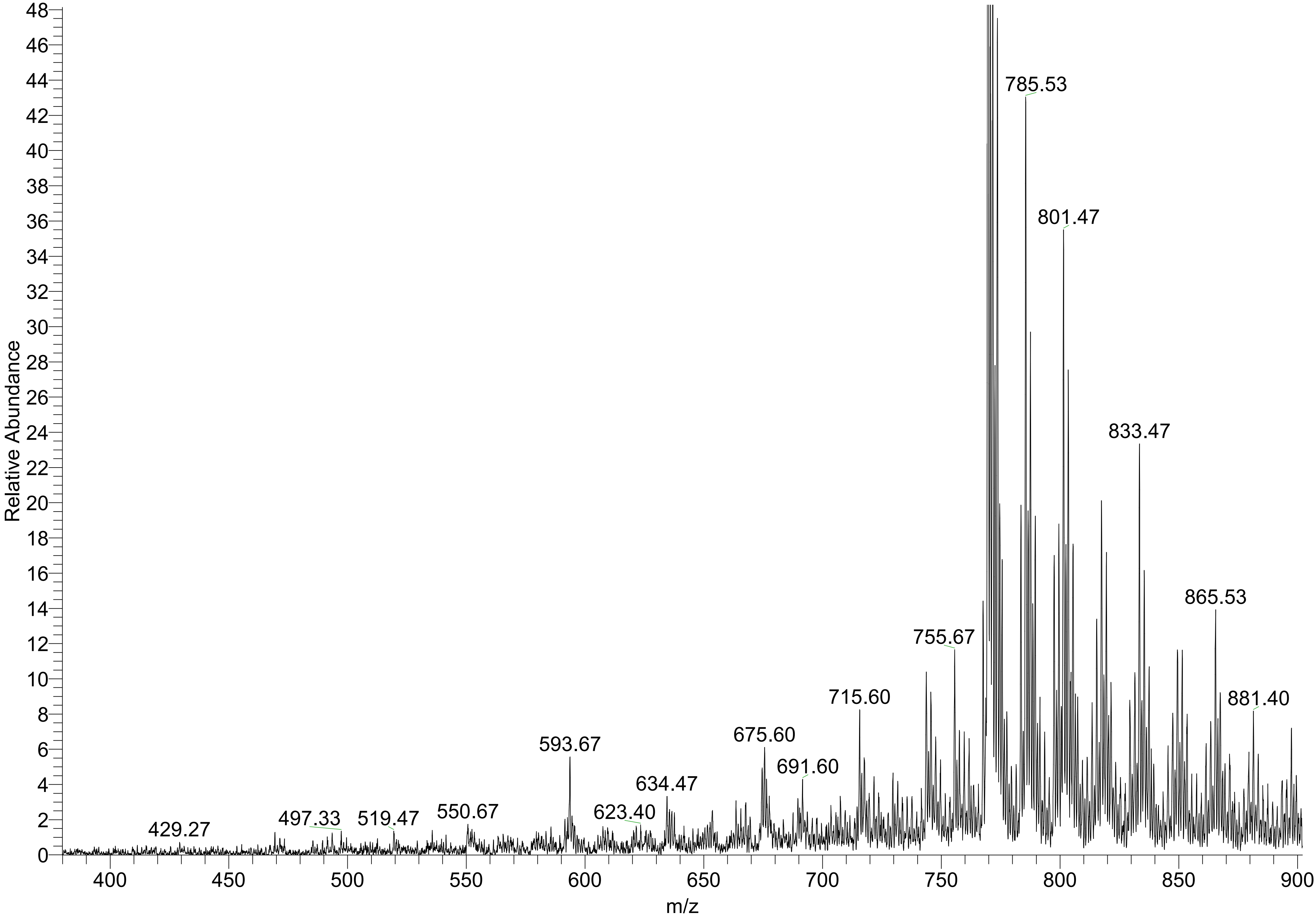

E:\dan_evans\RAW FILES\100%\CH8 08/16/18 14:16:06 CH8

CH8 #1-50 RT: 0.01-0.75 AV: 50 NL: 1.33E6
T: + p ESI ms [150.00-1000.00]

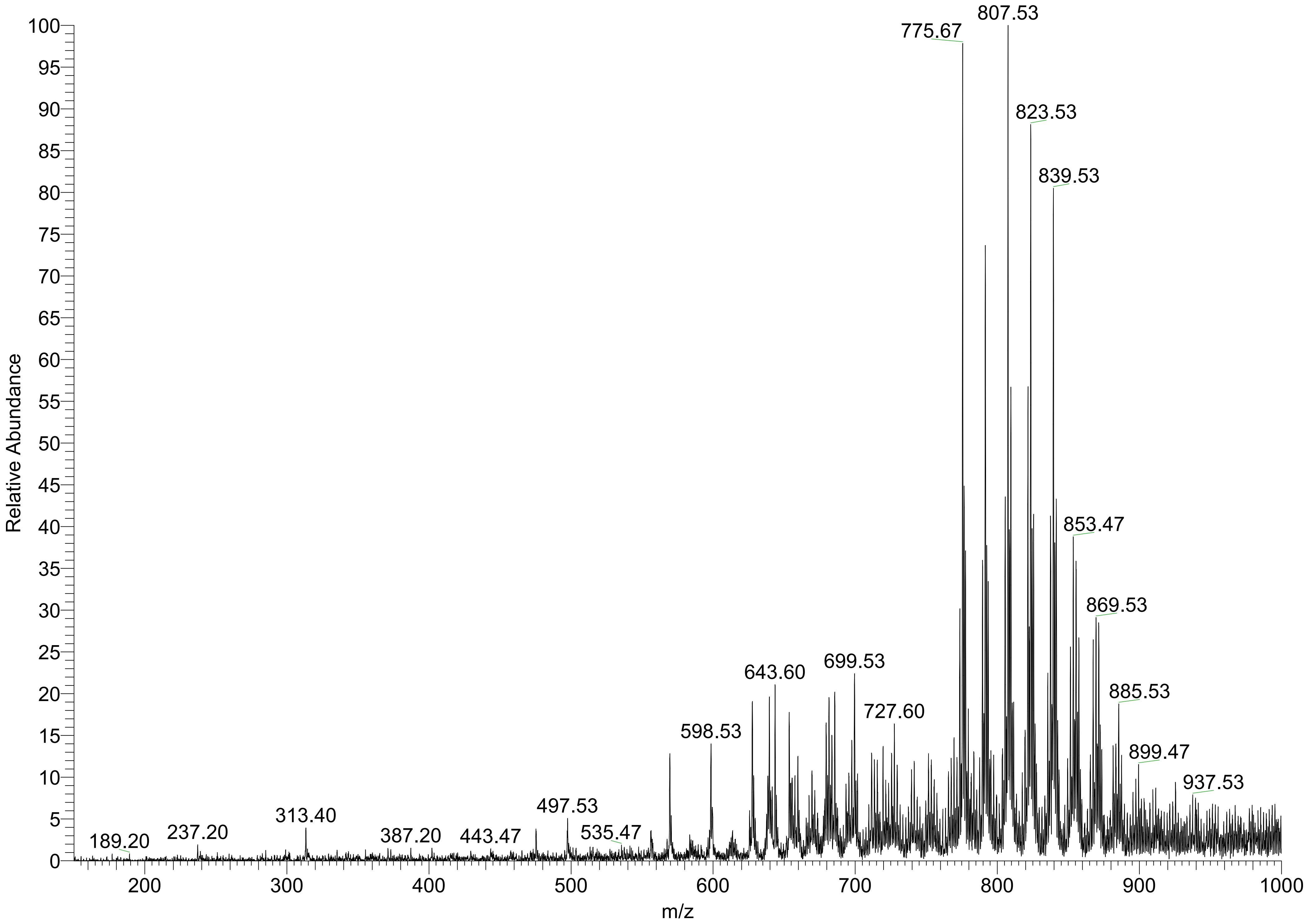

E:\dan_evans\RAW FILES\100%\CH8 08/16/18 14:16:06 CH8

CH8 #1-50 RT: 0.01-0.75 AV: 50 NL: 1.33E6
T: + p ESI ms [150.00-1000.00]

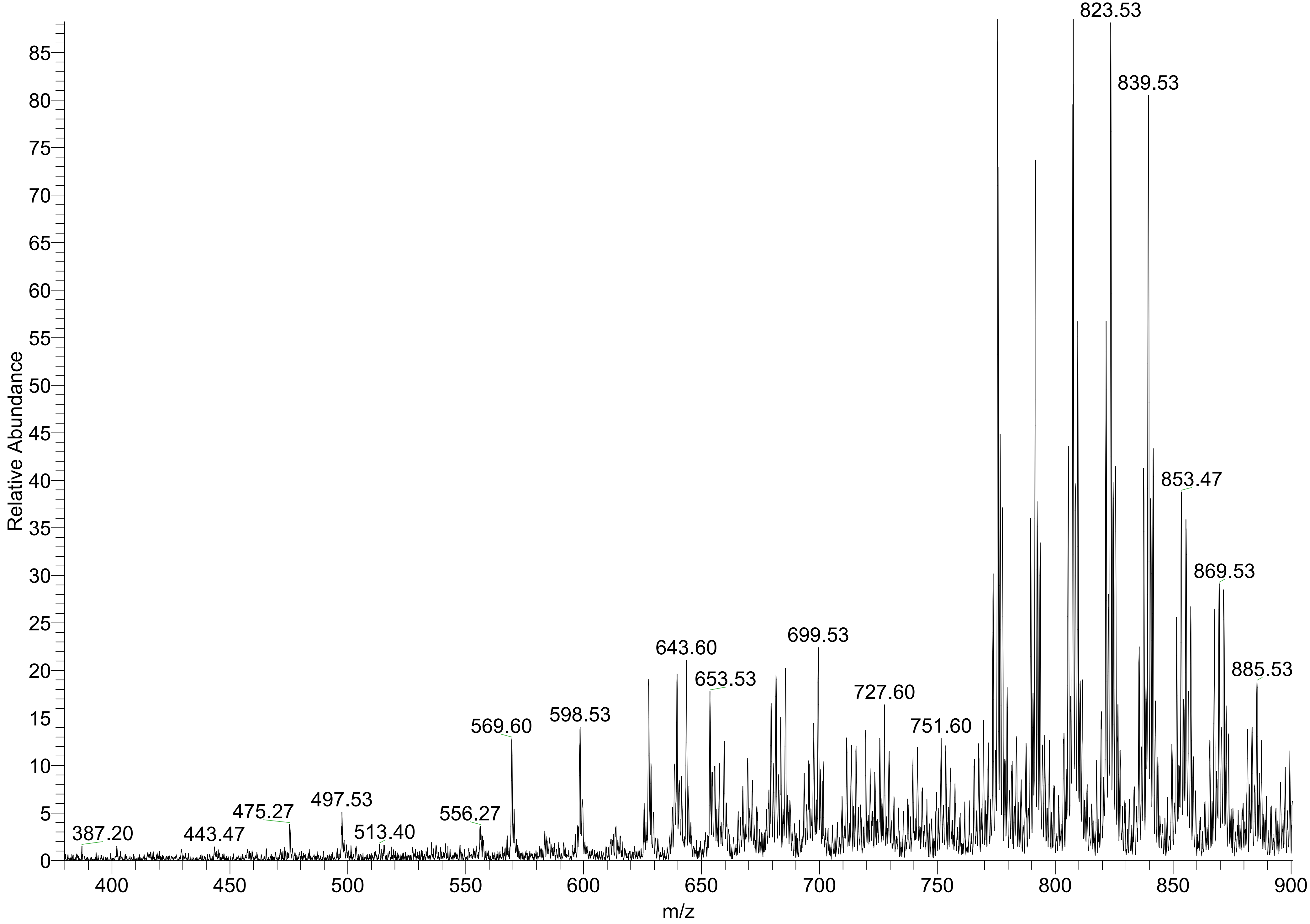

E:\dan_evans\RAW FILES\100%\CH9 08/16/18 14:21:16 CH9

CH9 #1-50 RT: 0.01-0.74 AV: 50 NL: 1.59E6
T: + p ESI ms [150.00-1000.00]

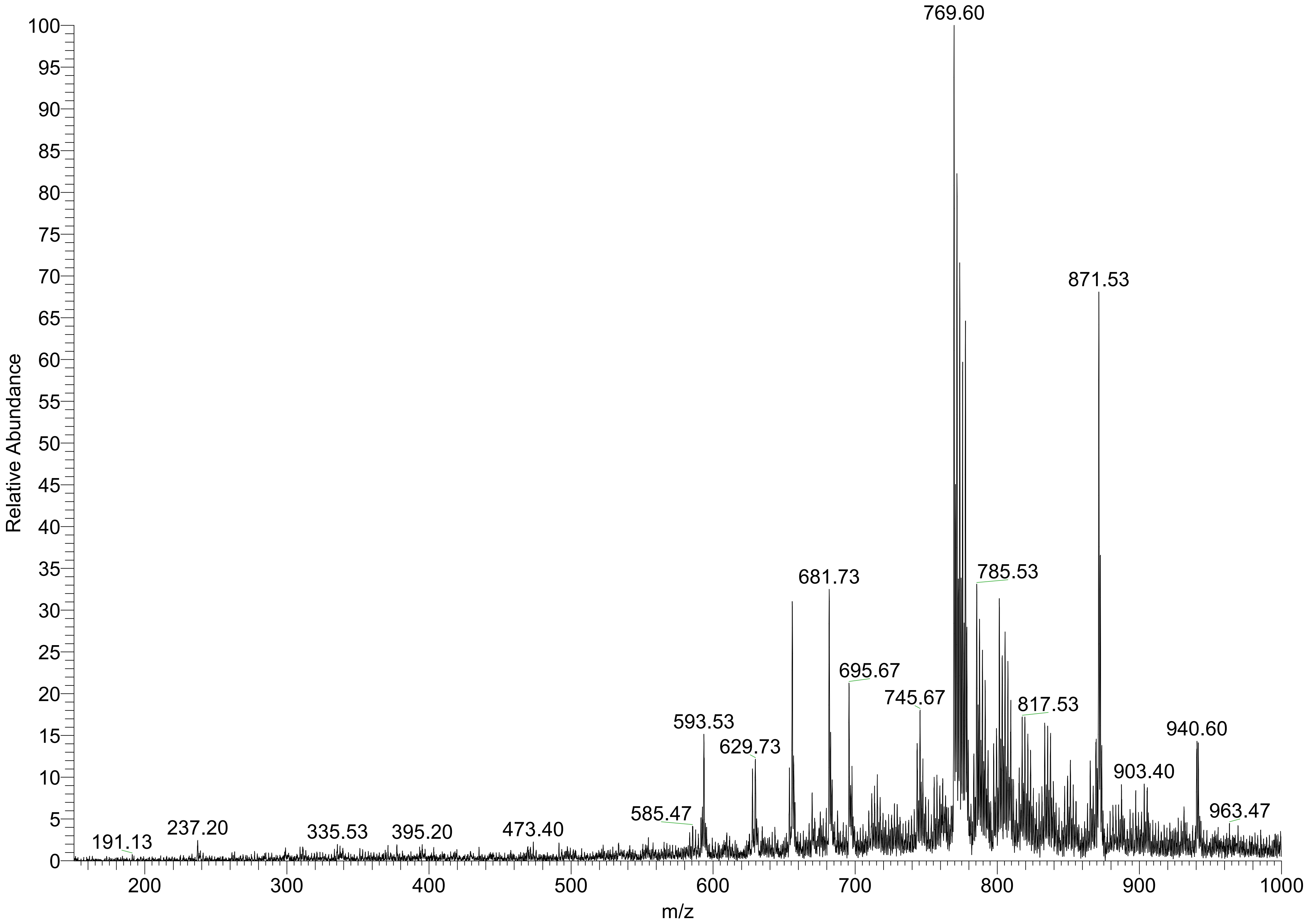

E:\dan_evans\RAW FILES\100%\CH9 08/16/18 14:21:16 CH9

CH9 #1-50 RT: 0.01-0.74 AV: 50 NL: 1.59E6
T: + p ESI ms [150.00-1000.00]

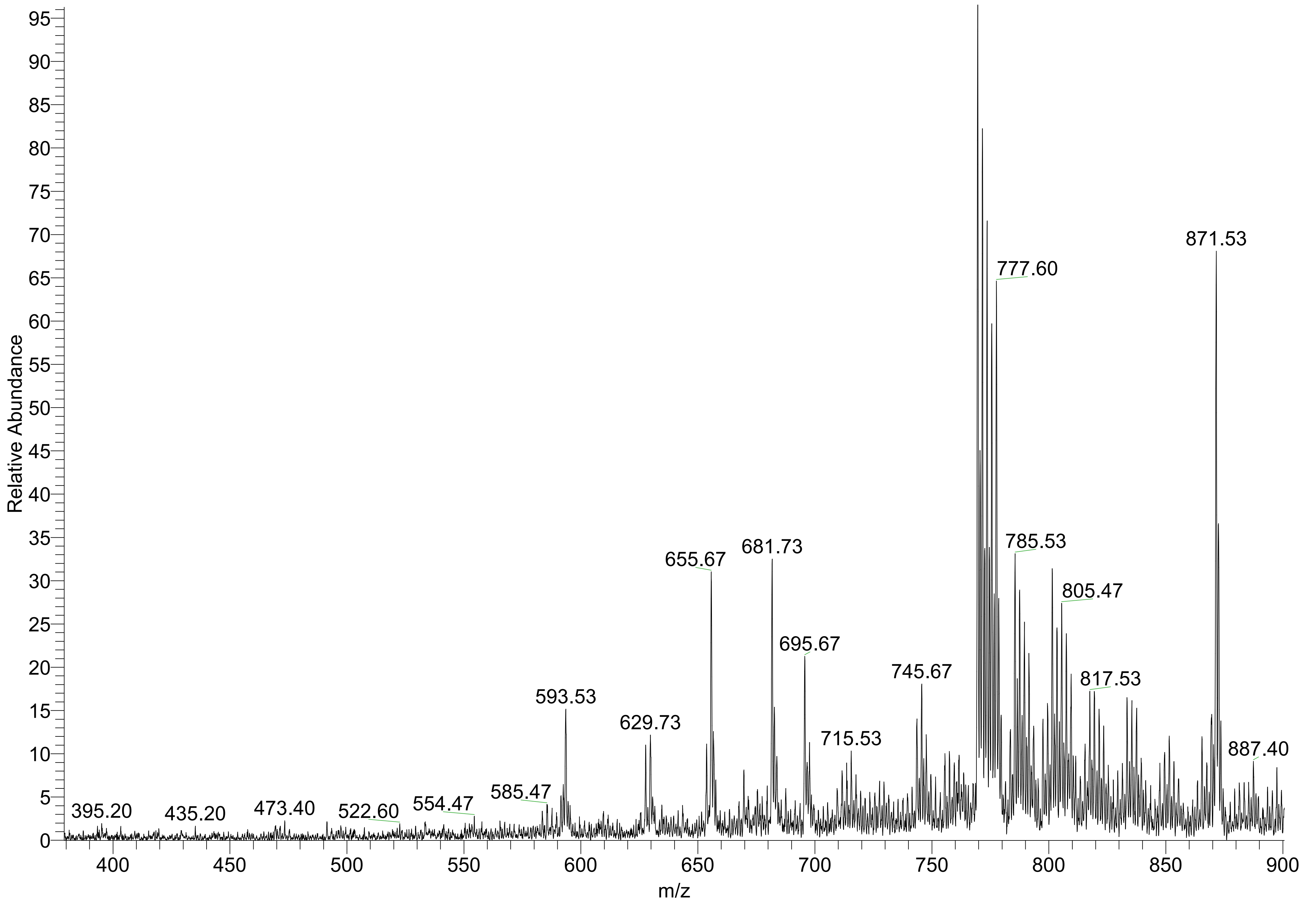

E:\dan_evans\RAW FILES\100%\CH10 08/16/18 14:29:32 CH10

CH10 #1-50 RT: 0.00-0.74 AV: 50 NL: 2.55E5
T: + p ESI ms [150.00-1000.00]

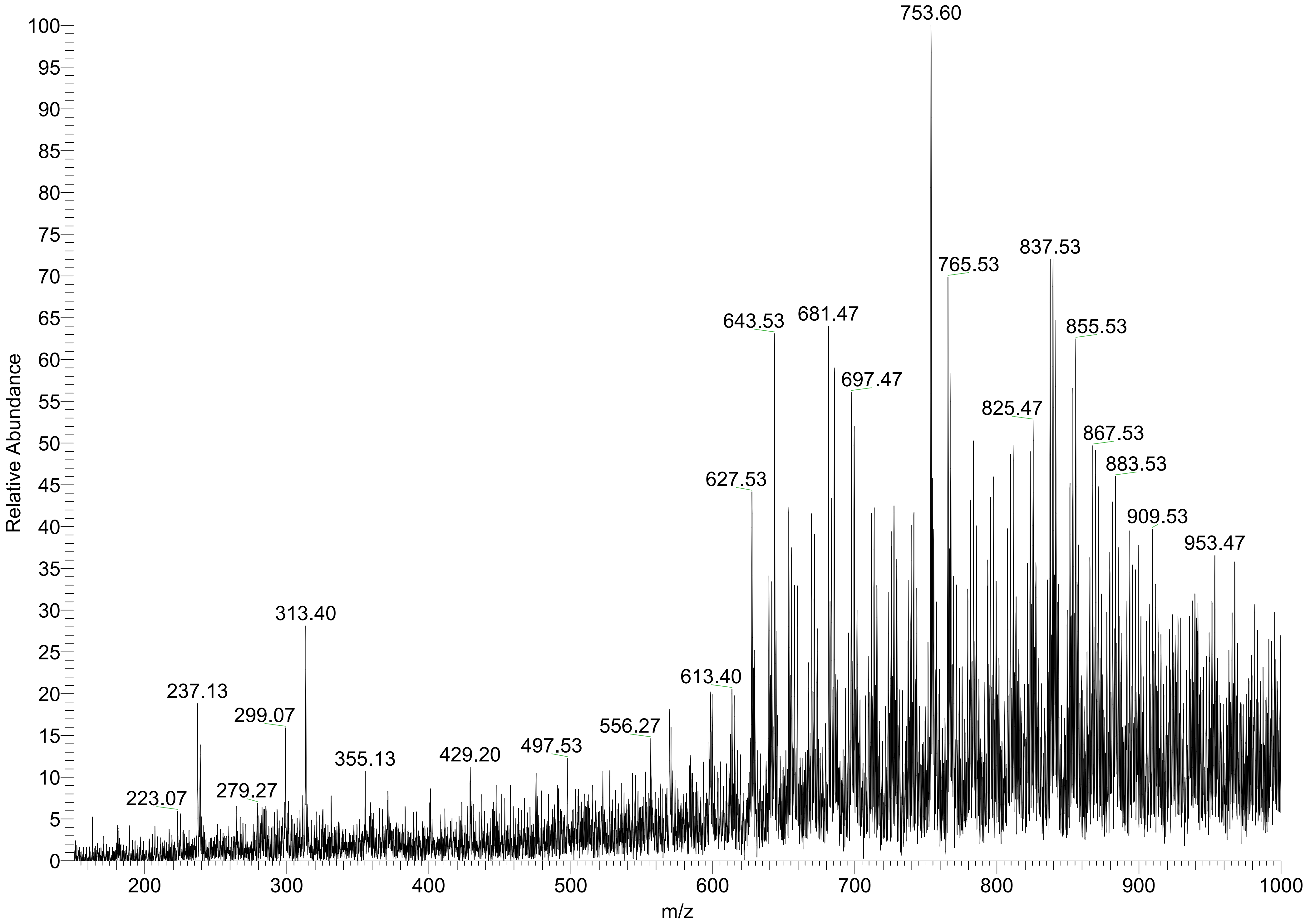

E:\dan_evans\RAW FILES\100%\CH10 08/16/18 14:29:32 CH10

CH10 #1-50 RT: 0.00-0.74 AV: 50 NL: 2.55E5
T: + p ESI ms [150.00-1000.00]

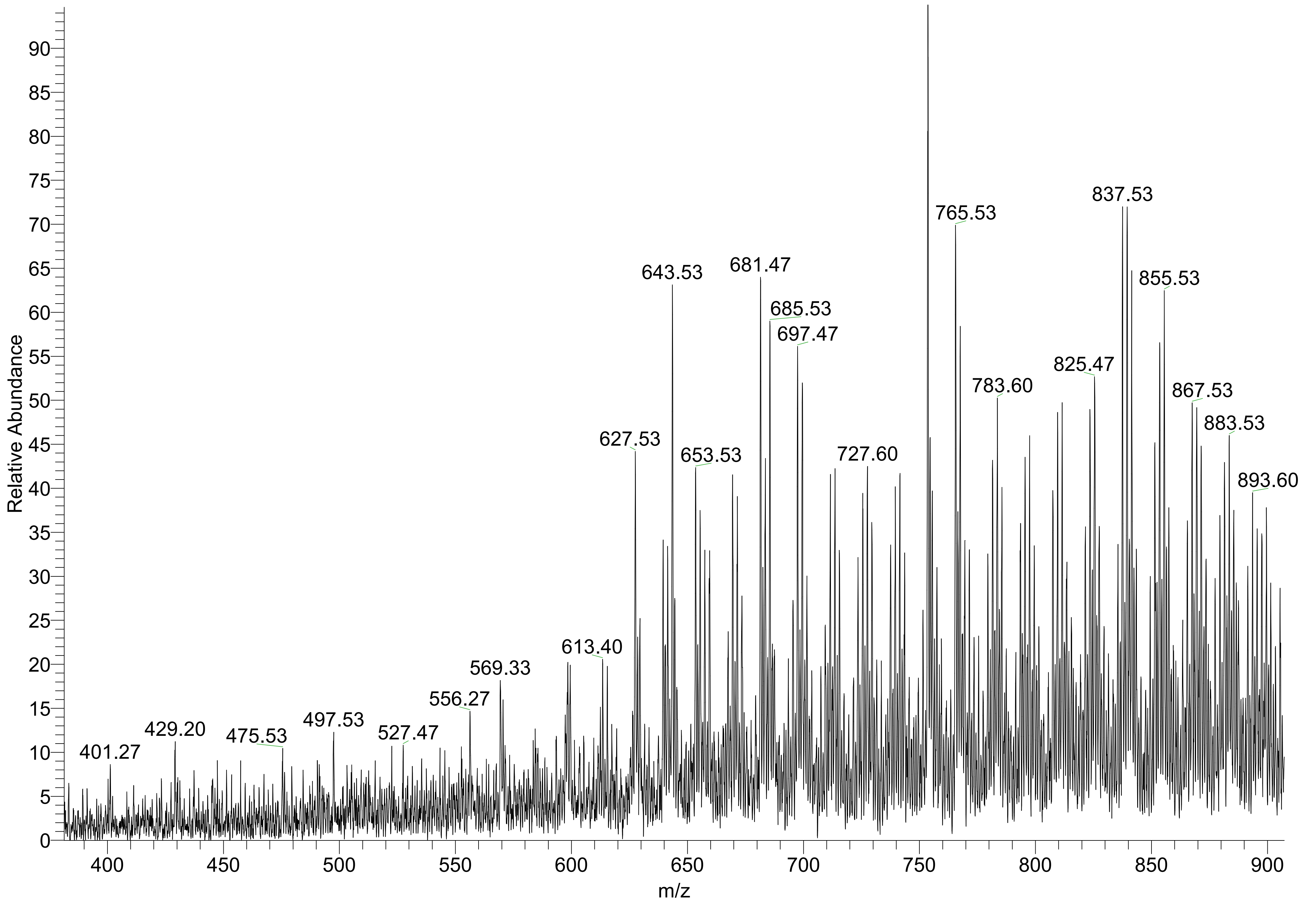

E:\dan_evans\RAW FILES\100%\S1 08/16/18 14:46:51 S1

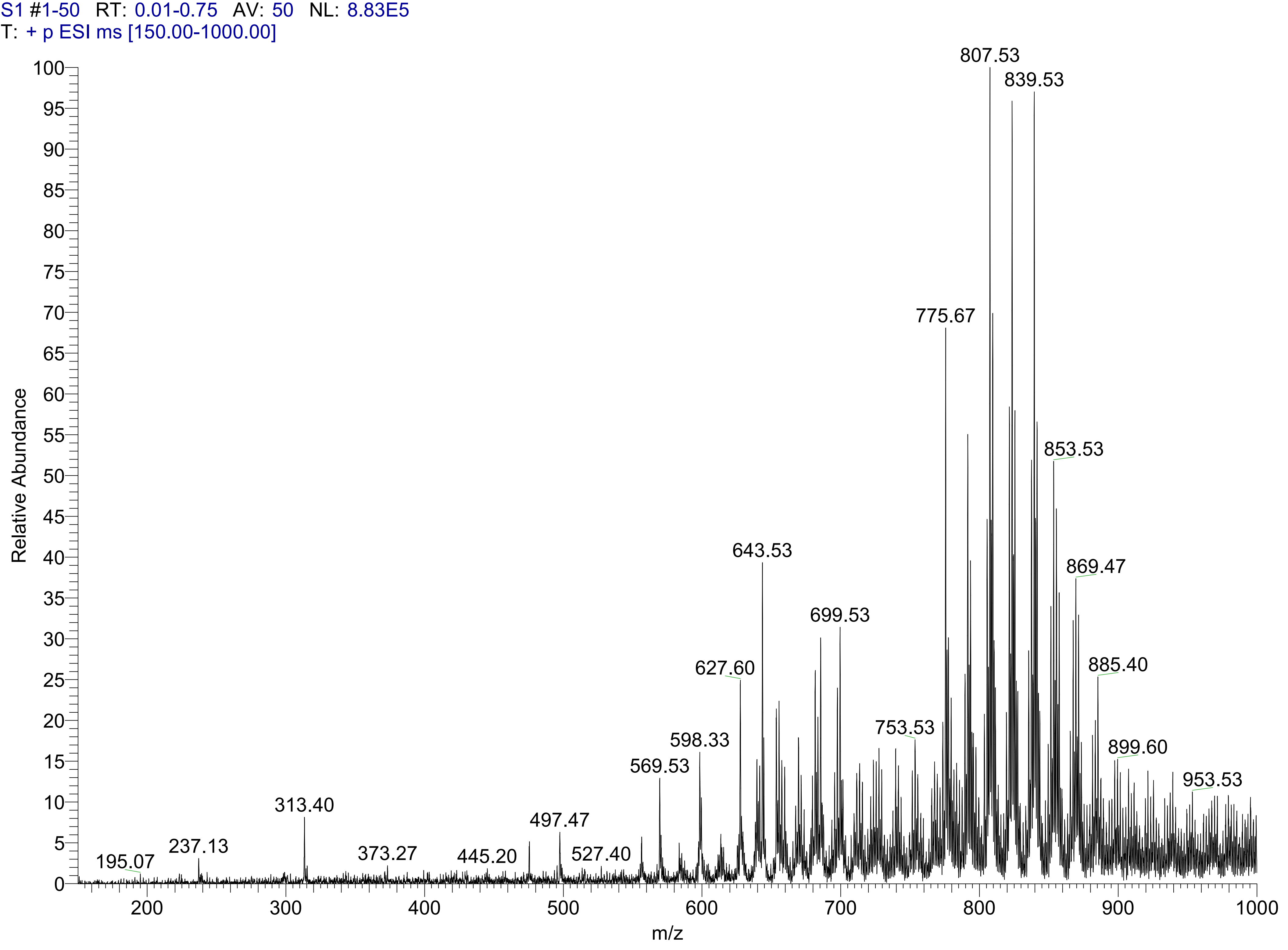

E:\dan_evans\RAW FILES\100%\S1 08/16/18 14:46:51 S1

S1 #1-50 RT: 0.01-0.75 AV: 50 NL: 8.83E5
T: + p ESI ms [150.00-1000.00]

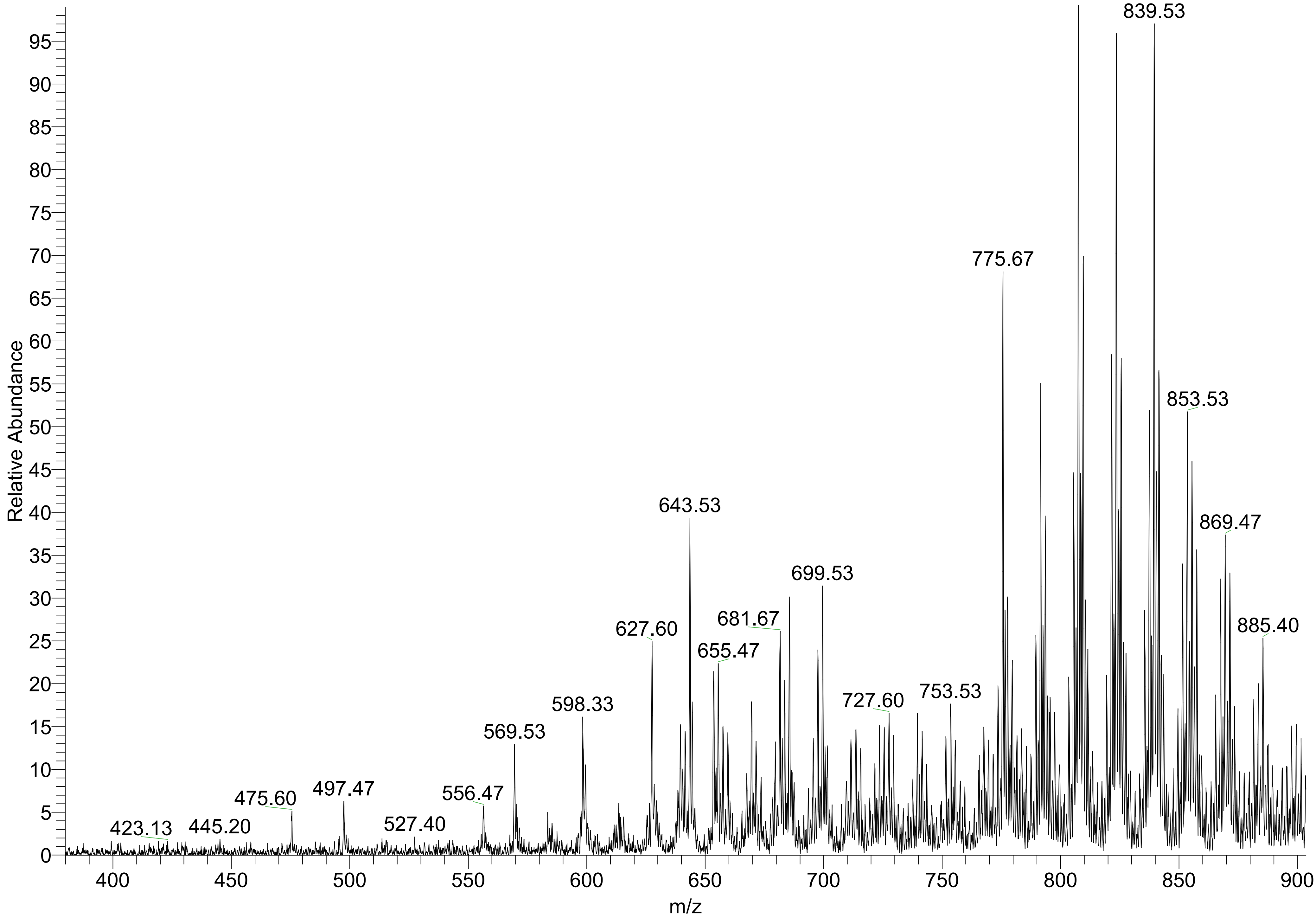

E:\dan_evans\RAW FILES\100%\S2 08/16/18 14:51:59 S2

S2 #1-50 RT: 0.00-0.74 AV: 50 NL: 5.99E5
T: + p ESI ms [150.00-1000.00]

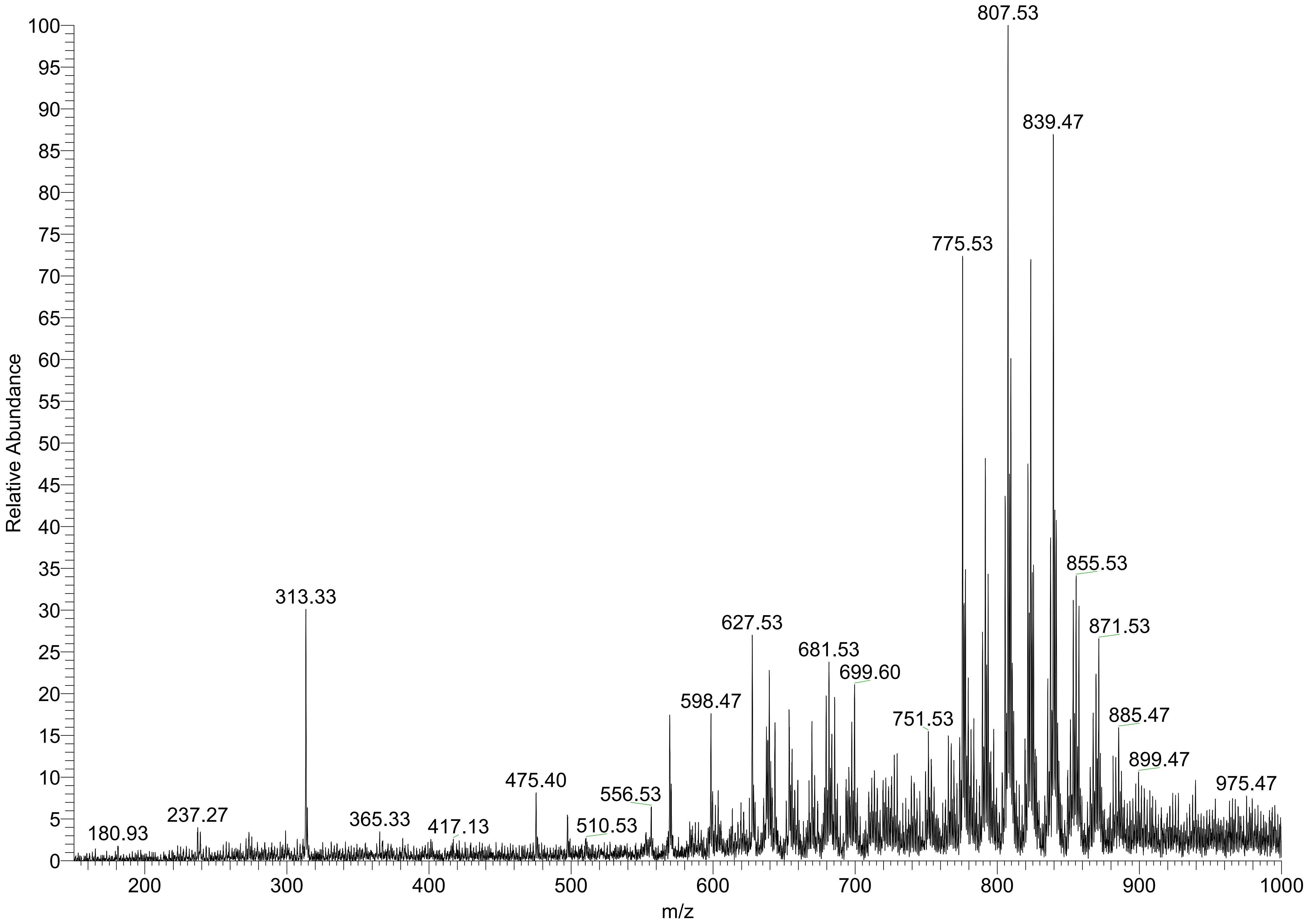

E:\dan_evans\RAW FILES\100%\S2 08/16/18 14:51:59 S2

S2 #1-50 RT: 0.00-0.74 AV: 50 NL: 5.99E5
T: + p ESI ms [150.00-1000.00]

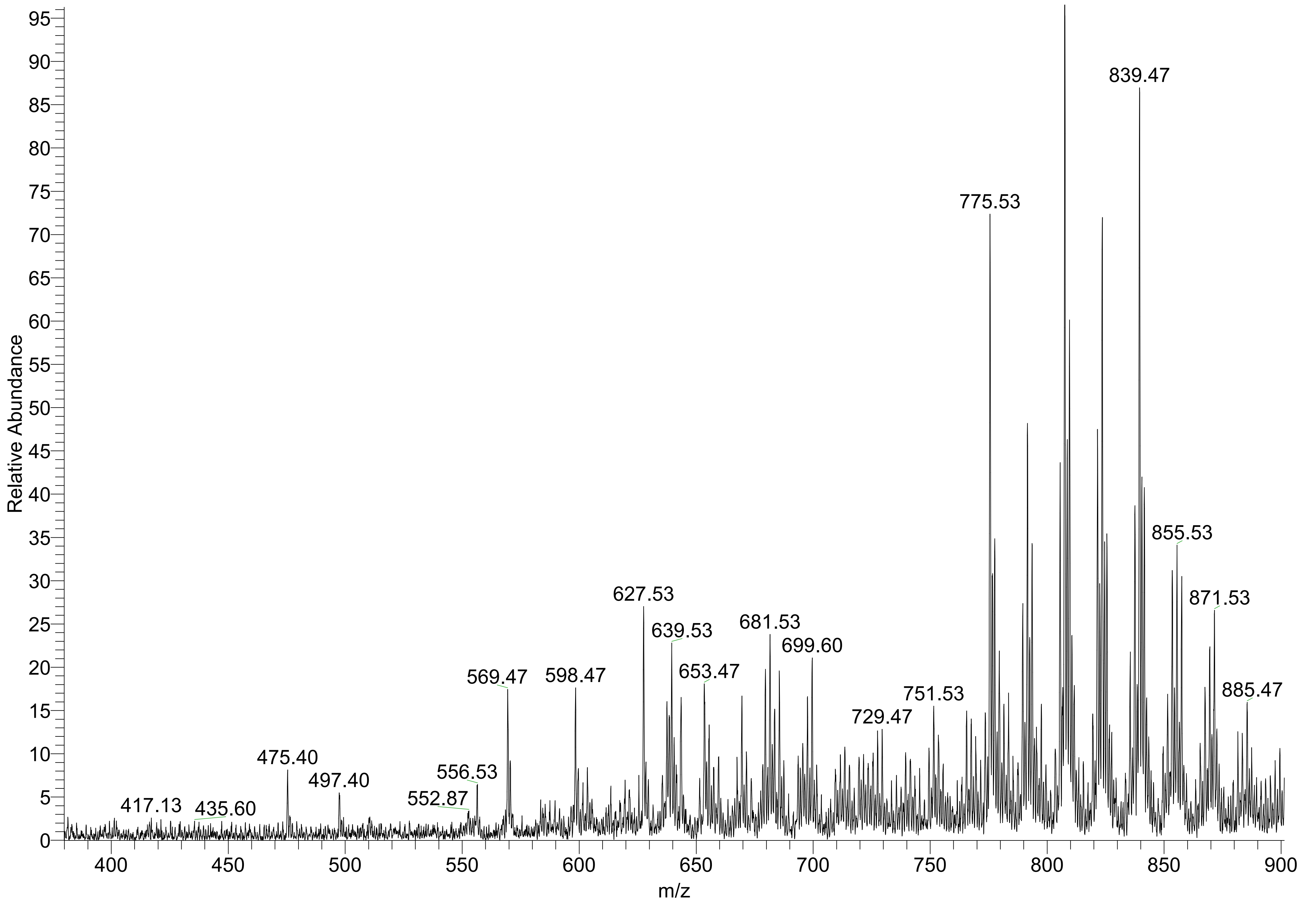

E:\dan_evans\RAW FILES\100%\S3 08/16/18 14:56:45 S3

S3 #1-50 RT: 0.01-0.74 AV: 50 NL: 9.38E5
T: + p ESI ms [150.00-1000.00]

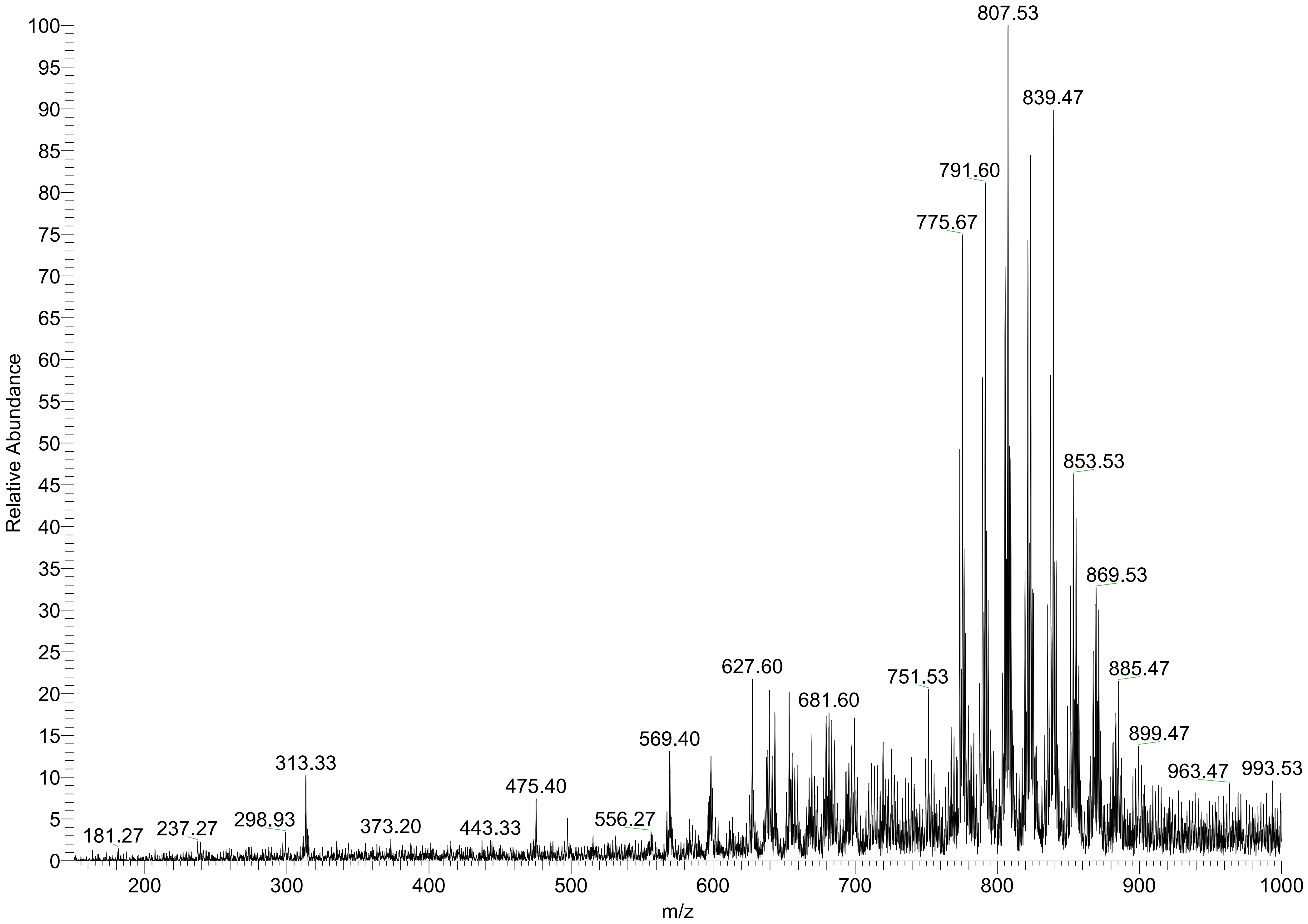

E:\dan_evans\RAW FILES\100%\S3 08/16/18 14:56:45 S3

S3 #1-50 RT: 0.01-0.74 AV: 50 NL: 9.38E5
T: + p ESI ms [150.00-1000.00]

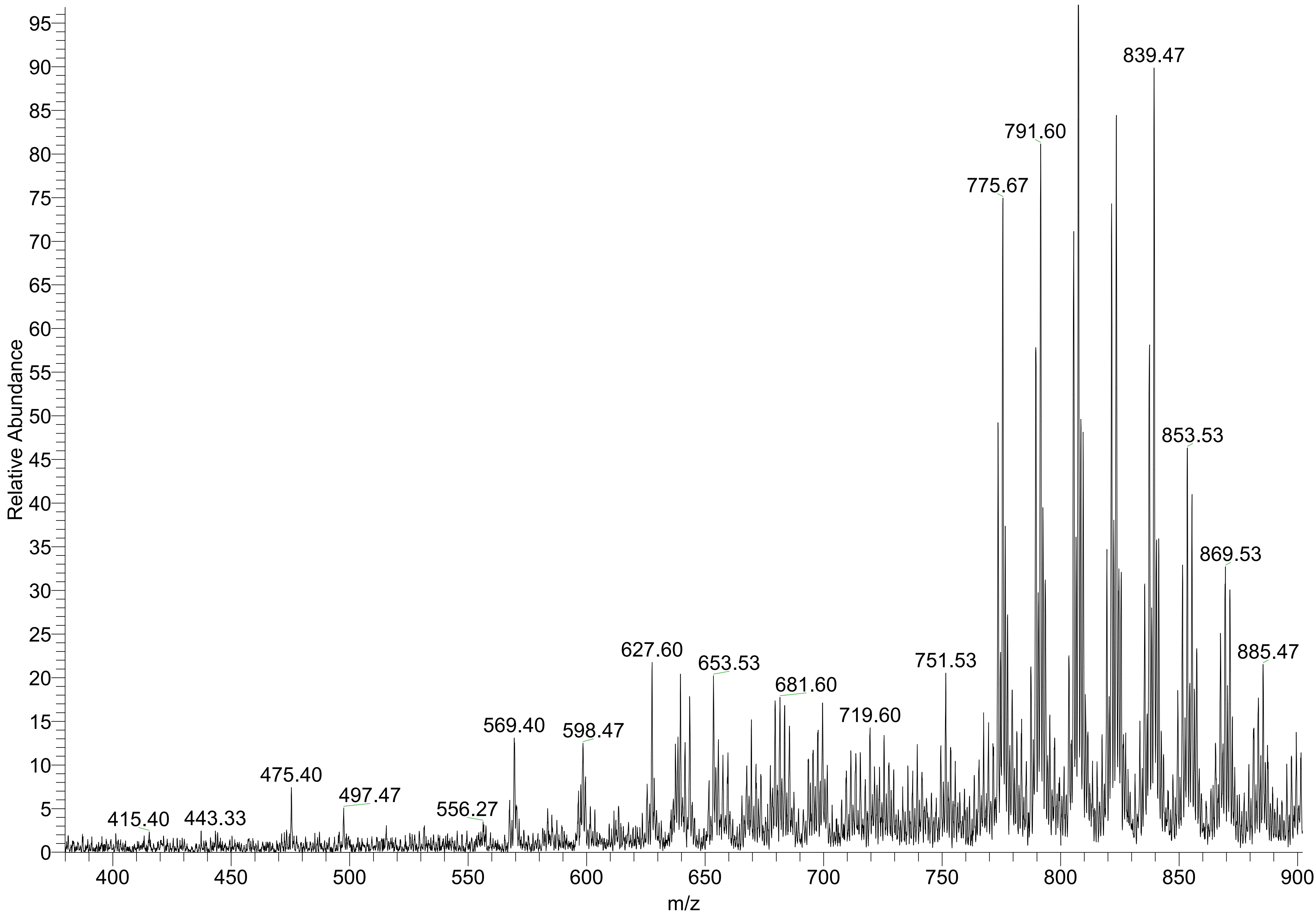

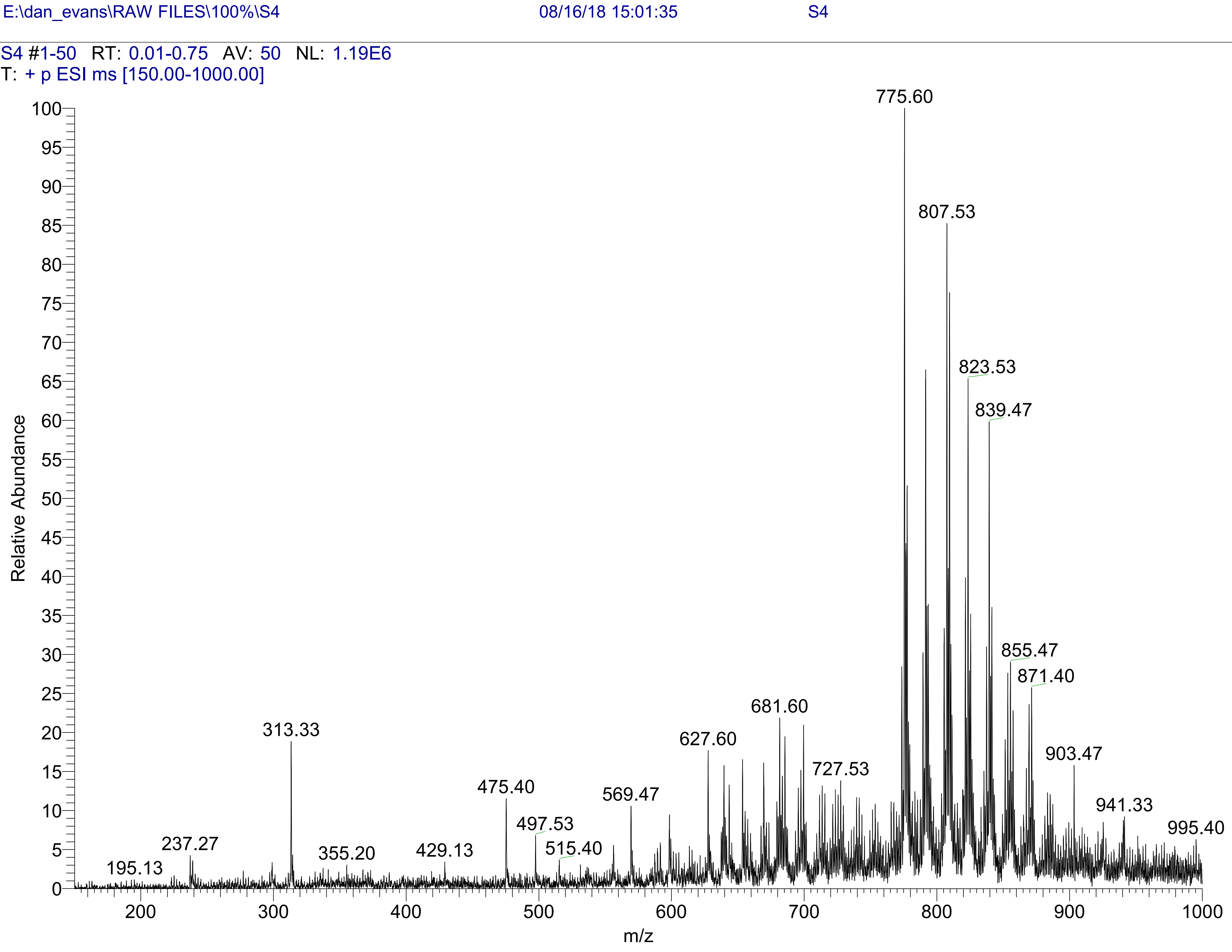

E:\dan_evans\RAW FILES\100%\S4
08/16/18 15:01:35
S4
S4 #1-50 RT: 0.01-0.75 AV: 50 NL: 1.19E6
T: + p ESI ms [150.00-1000.00]
775.60
807.53
823.53
839.47
855.47
871.40
903.47
941.33
995.40
681.60
627.60
727.53
569.47
475.40
497.53
515.40
429.13
355.20
313.33
237.27
195.13
Relative Abundance
m/z

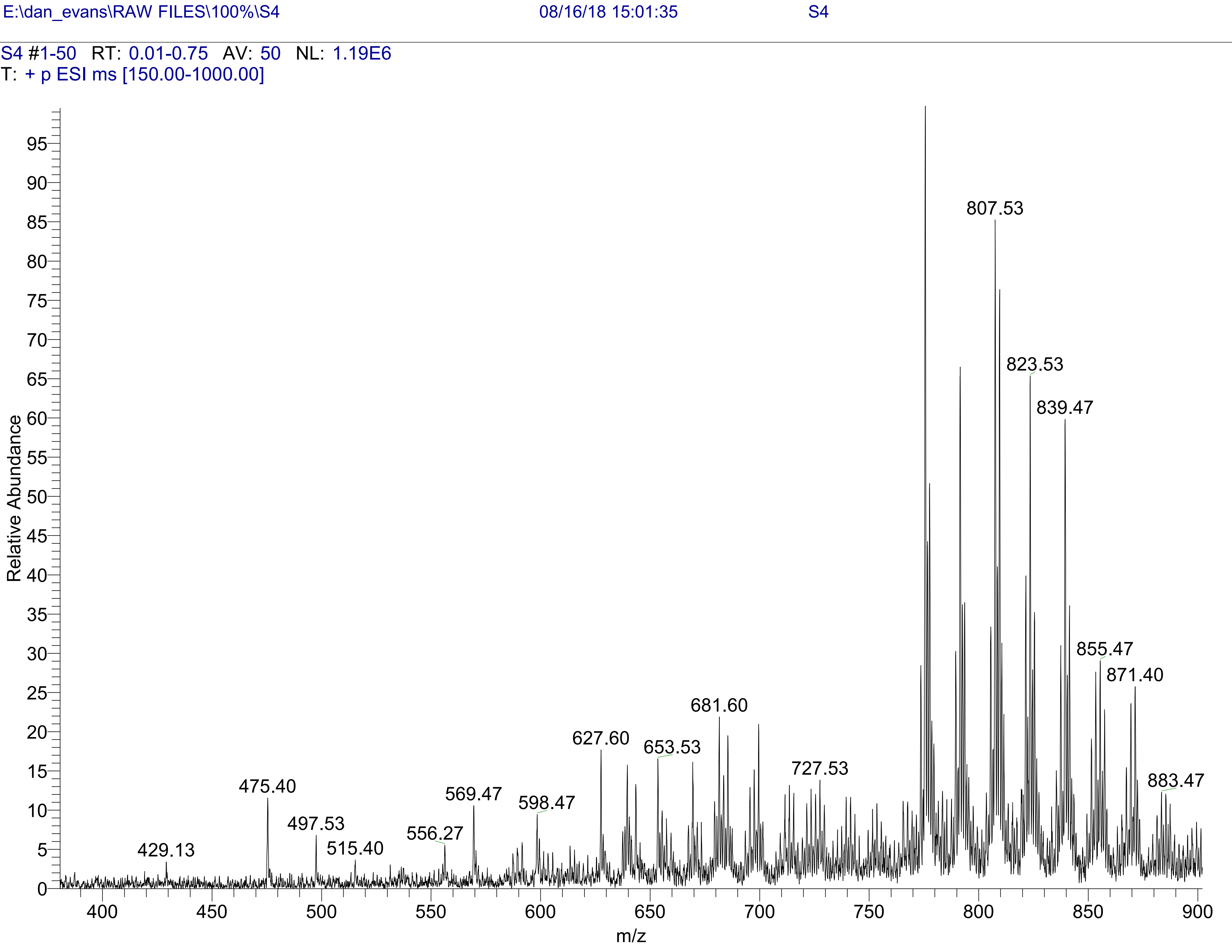
E:\dan_evans\RAW FILES\100%\S4
08/16/18 15:01:35
S4
S4 #1-50 RT: 0.01-0.75 AV: 50 NL: 1.19E6
T: + p ESI ms [150.00-1000.00]
Relative Abundance
m/z
807.53
823.53
839.47
855.47
871.40
883.47
681.60
627.60
653.53
727.53
475.40
569.47
598.47
497.53
556.27
515.40
429.13

E:\dan_evans\RAW FILES\100%\S5 08/16/18 15:06:39 S5

S5 #1-50 RT: 0.01-0.75 AV: 50 NL: 9.50E5
T: + p ESI ms [150.00-1000.00]

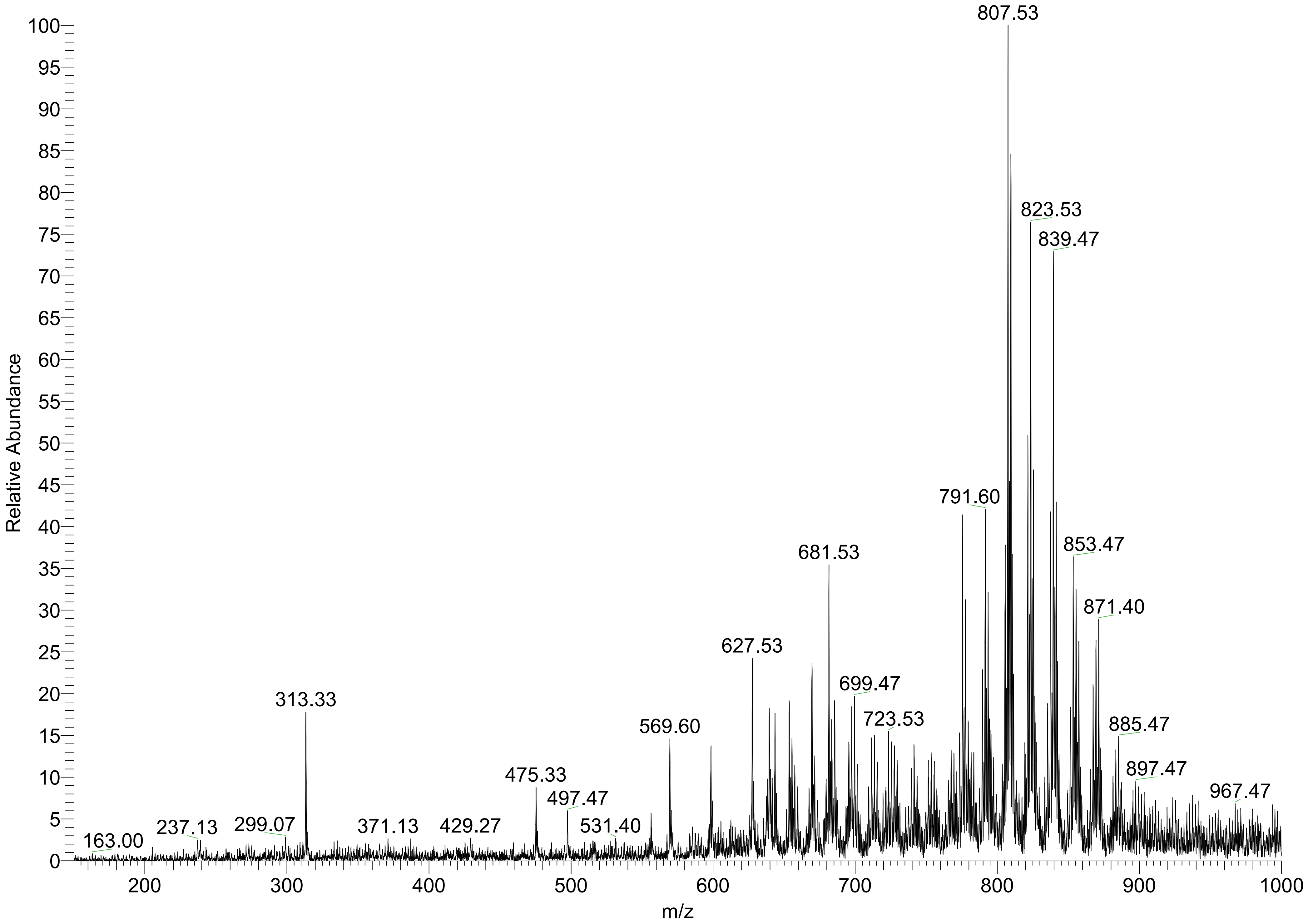

E:\dan_evans\RAW FILES\100%\S5 08/16/18 15:06:39 S5

S5 #1-50 RT: 0.01-0.75 AV: 50 NL: 9.50E5
T: + p ESI ms [150.00-1000.00]

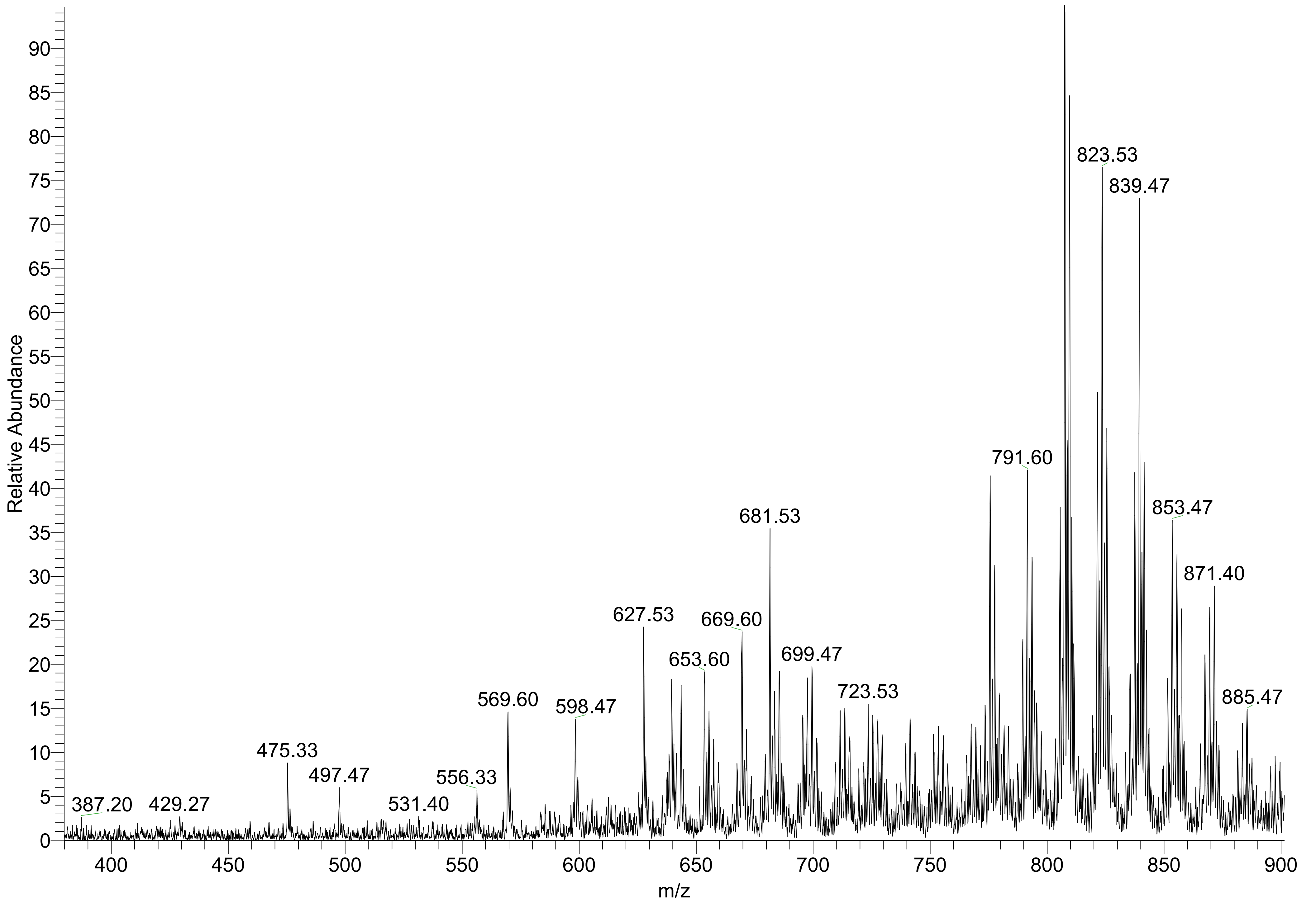

E:\dan_evans\RAW FILES\100%\S6 08/16/18 15:11:09 S6

S6 #1-50 RT: 0.01-0.75 AV: 50 NL: 8.62E5
T: + p ESI ms [150.00-1000.00]

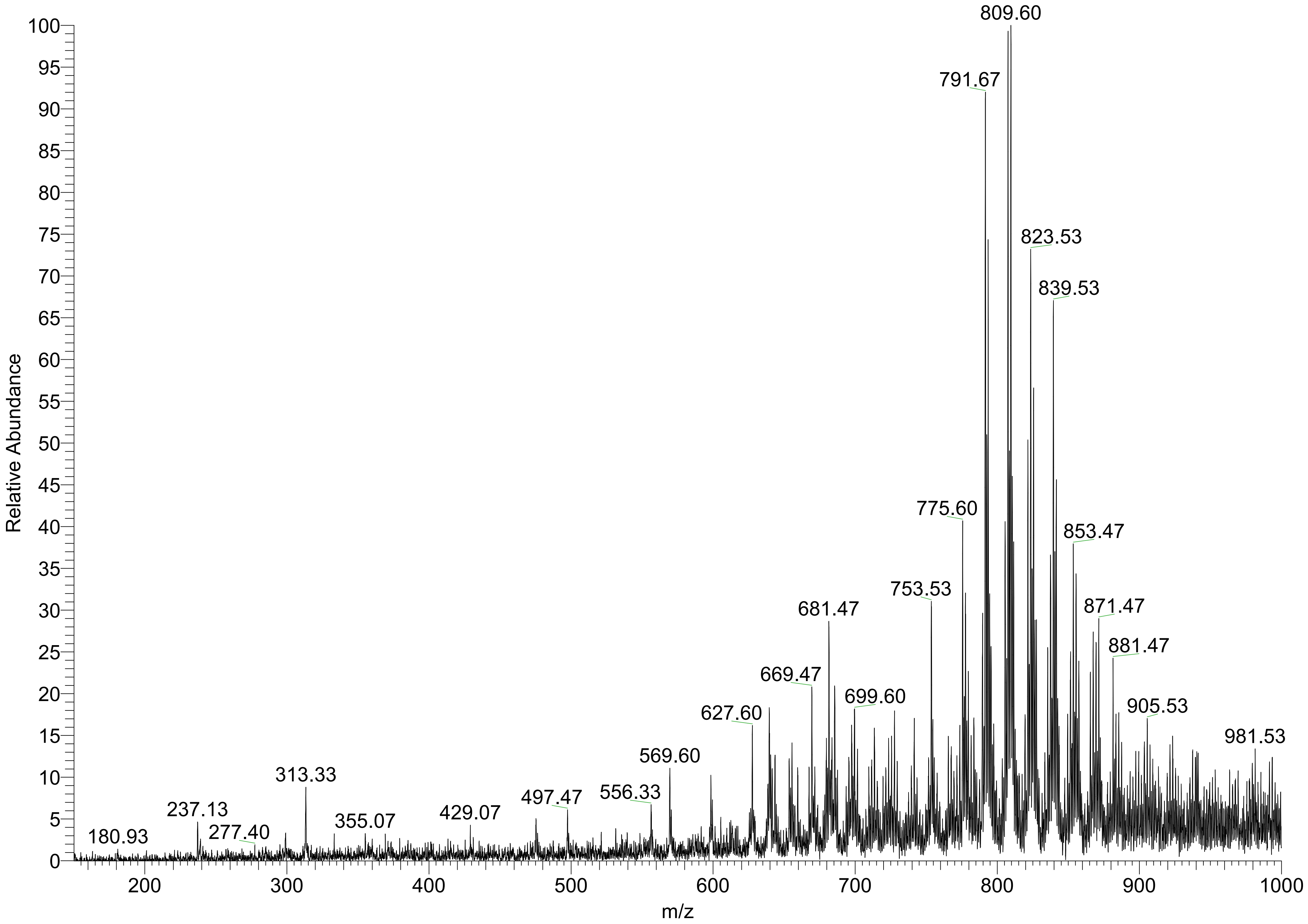

E:\dan_evans\RAW FILES\100%\S6 08/16/18 15:11:09 S6

S6 #1-50 RT: 0.01-0.75 AV: 50 NL: 8.62E5
T: + p ESI ms [150.00-1000.00]

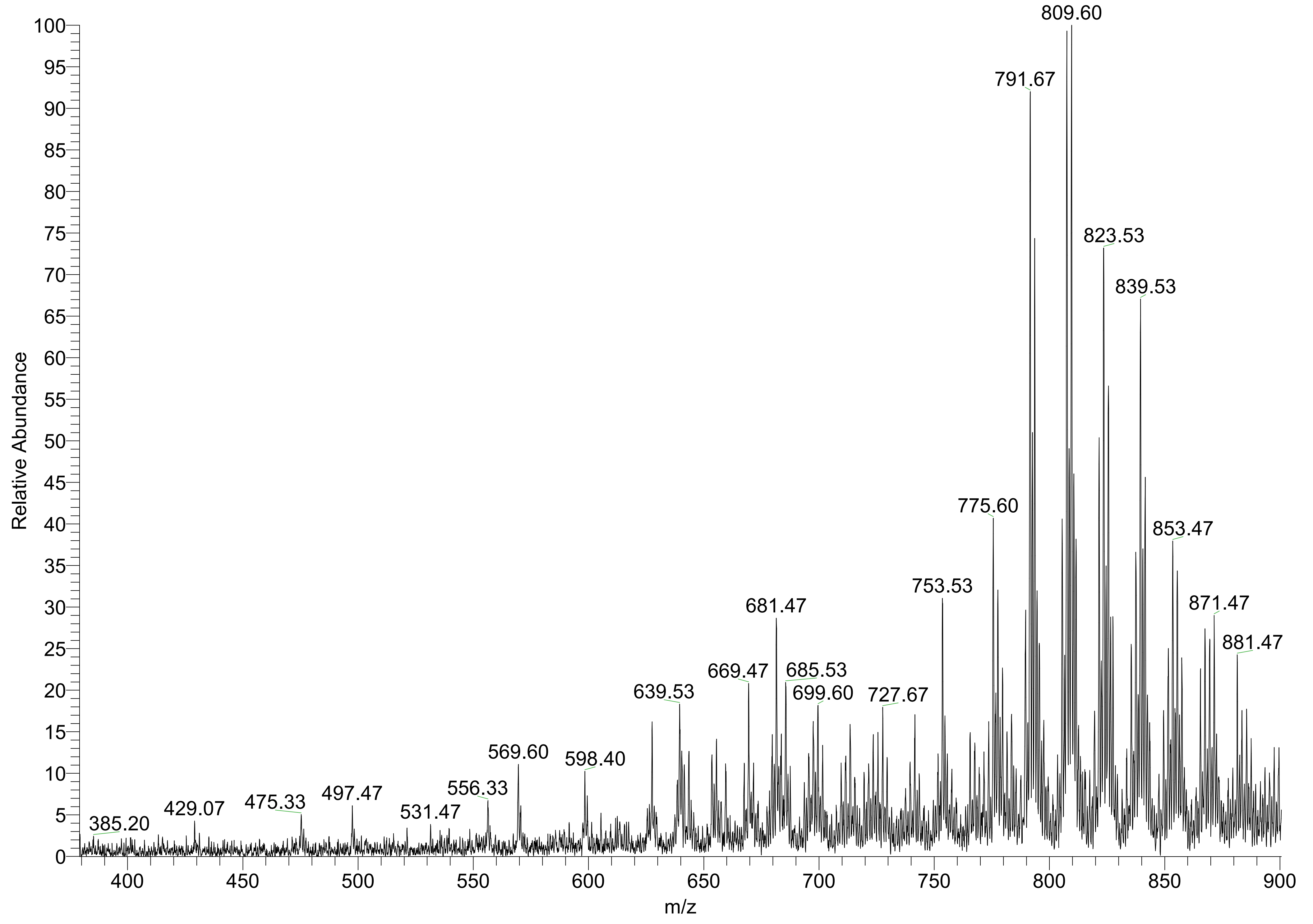

E:\dan_evans\RAW FILES\100%\S7 08/16/18 15:15:28 S7

S7 #1-50 RT: 0.01-0.74 AV: 50 NL: 8.18E5
T: + p ESI ms [150.00-1000.00]

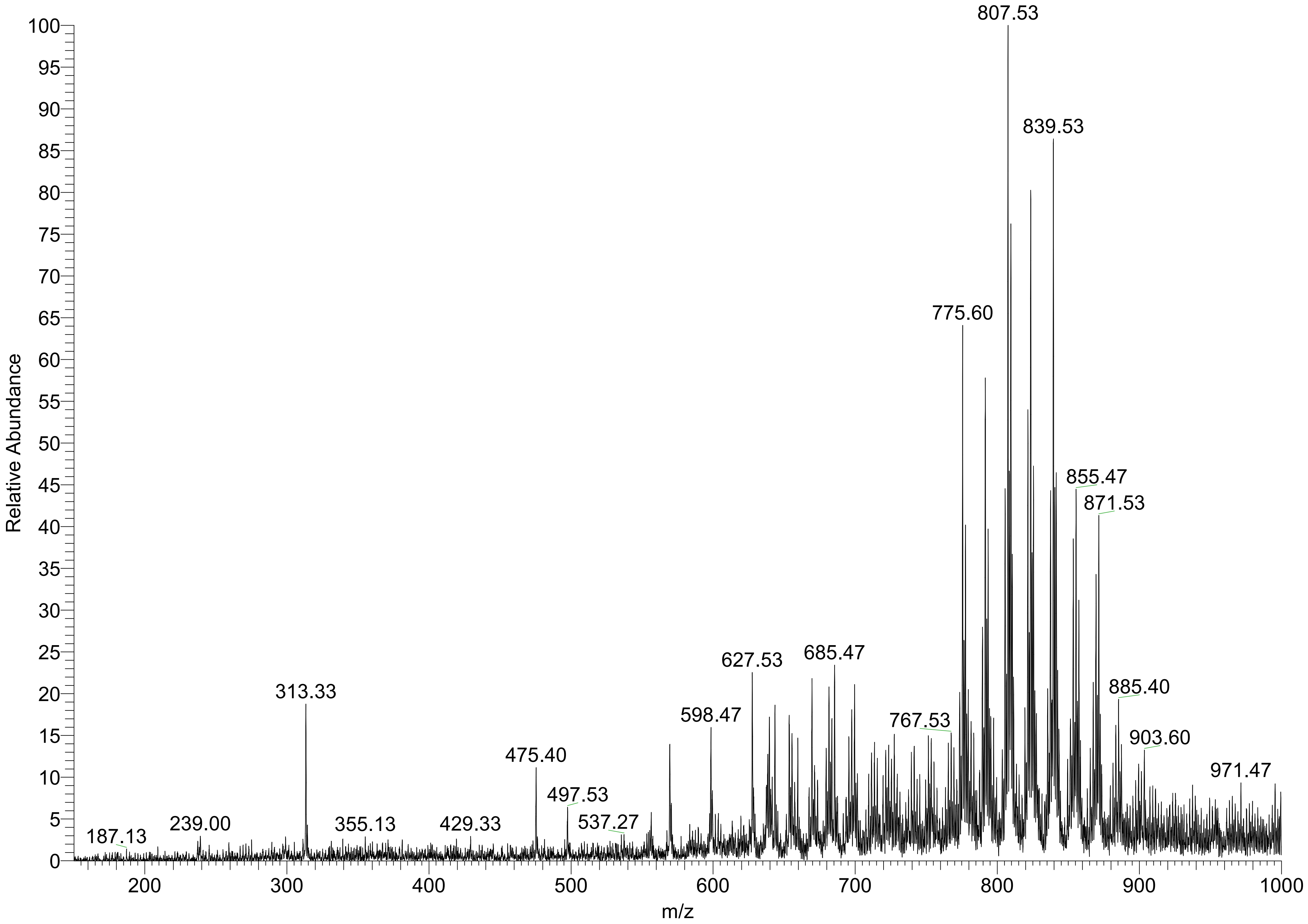

E:\dan_evans\RAW FILES\100%\S7 08/16/18 15:15:28 S7

S7 #1-50 RT: 0.01-0.74 AV: 50 NL: 8.18E5
T: + p ESI ms [150.00-1000.00]

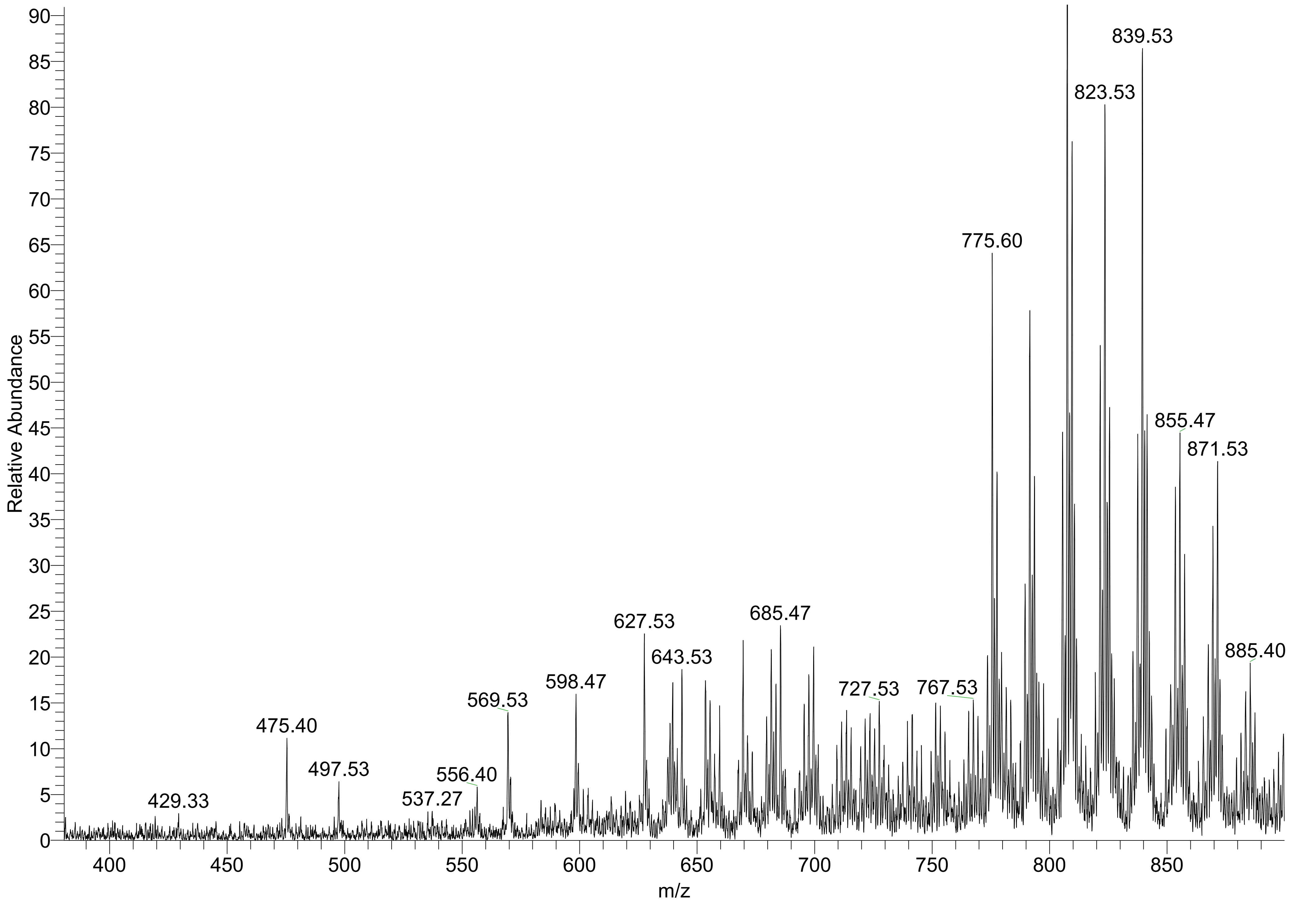

E:\dan_evans\RAW FILES\100%\S8 08/16/18 15:20:05 S8

S8 #1-50 RT: 0.01-0.74 AV: 50 NL: 7.94E5
T: + p ESI ms [150.00-1000.00]

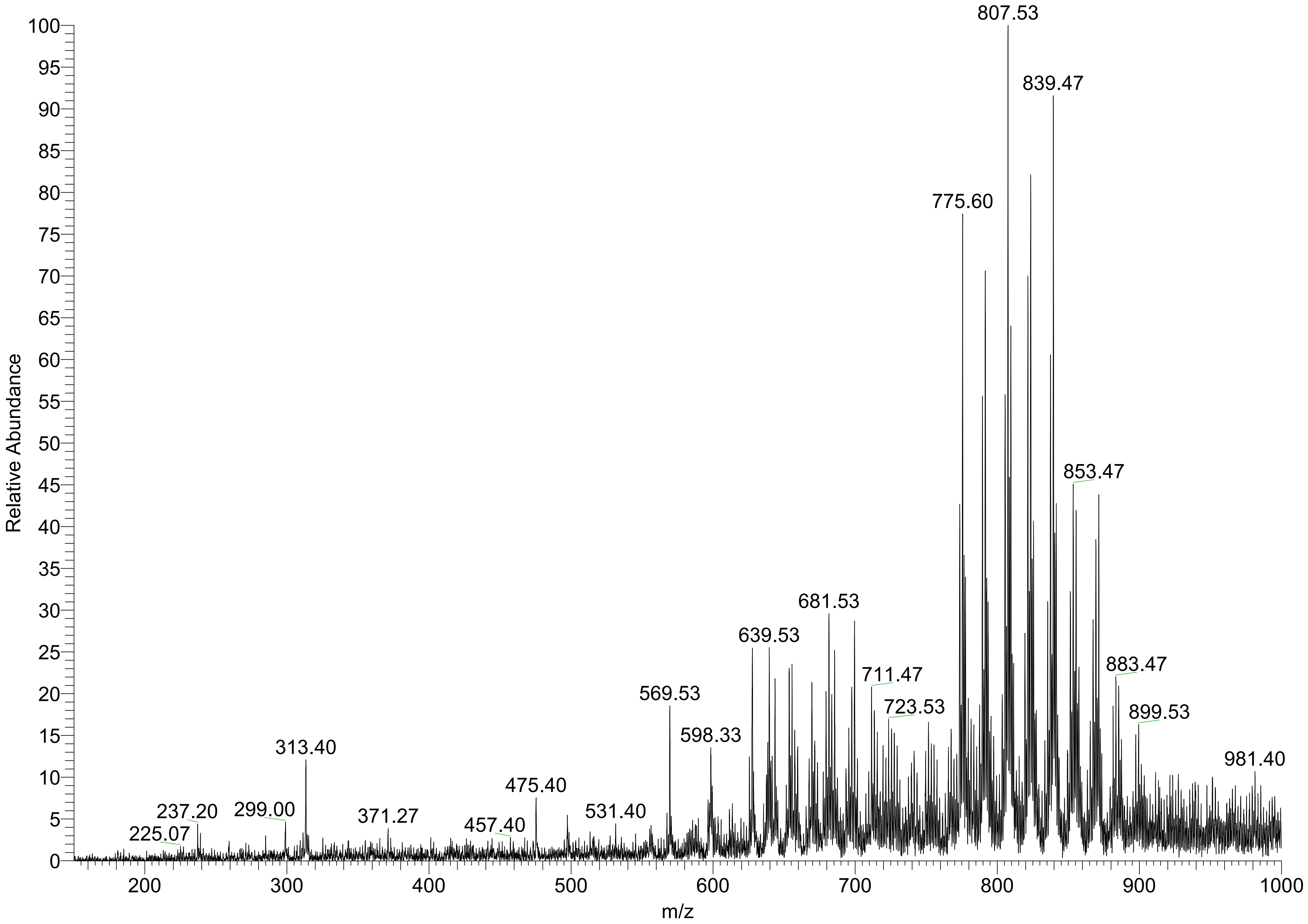

E:\dan_evans\RAW FILES\100%\S8 08/16/18 15:20:05 S8

S8 #1-50 RT: 0.01-0.74 AV: 50 NL: 7.94E5
T: + p ESI ms [150.00-1000.00]

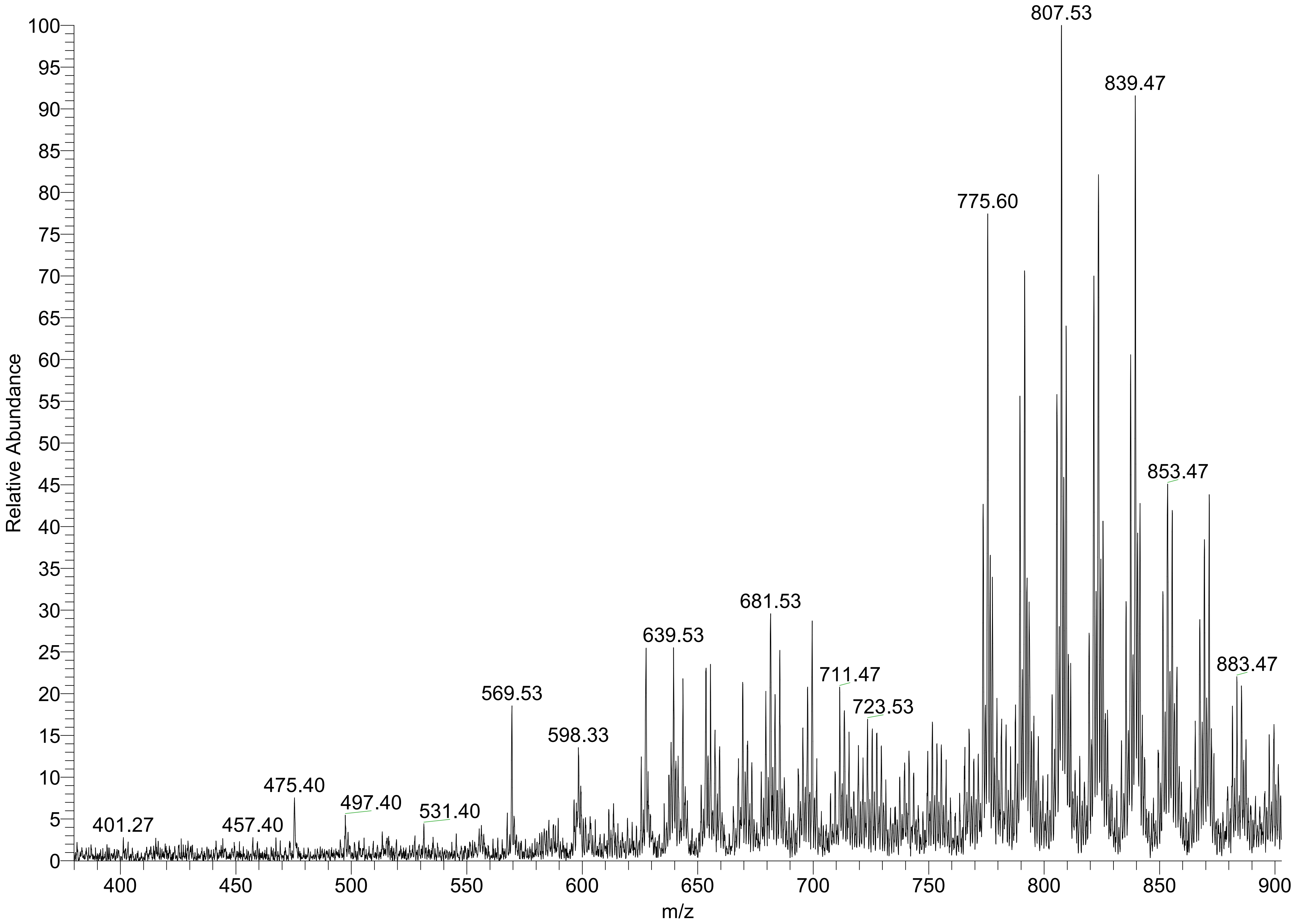

E:\dan_evans\RAW FILES\100%\S9 08/16/18 15:25:28 S9

S9 #1-50 RT: 0.01-0.75 AV: 50 NL: 7.68E5
T: + p ESI ms [150.00-1000.00]

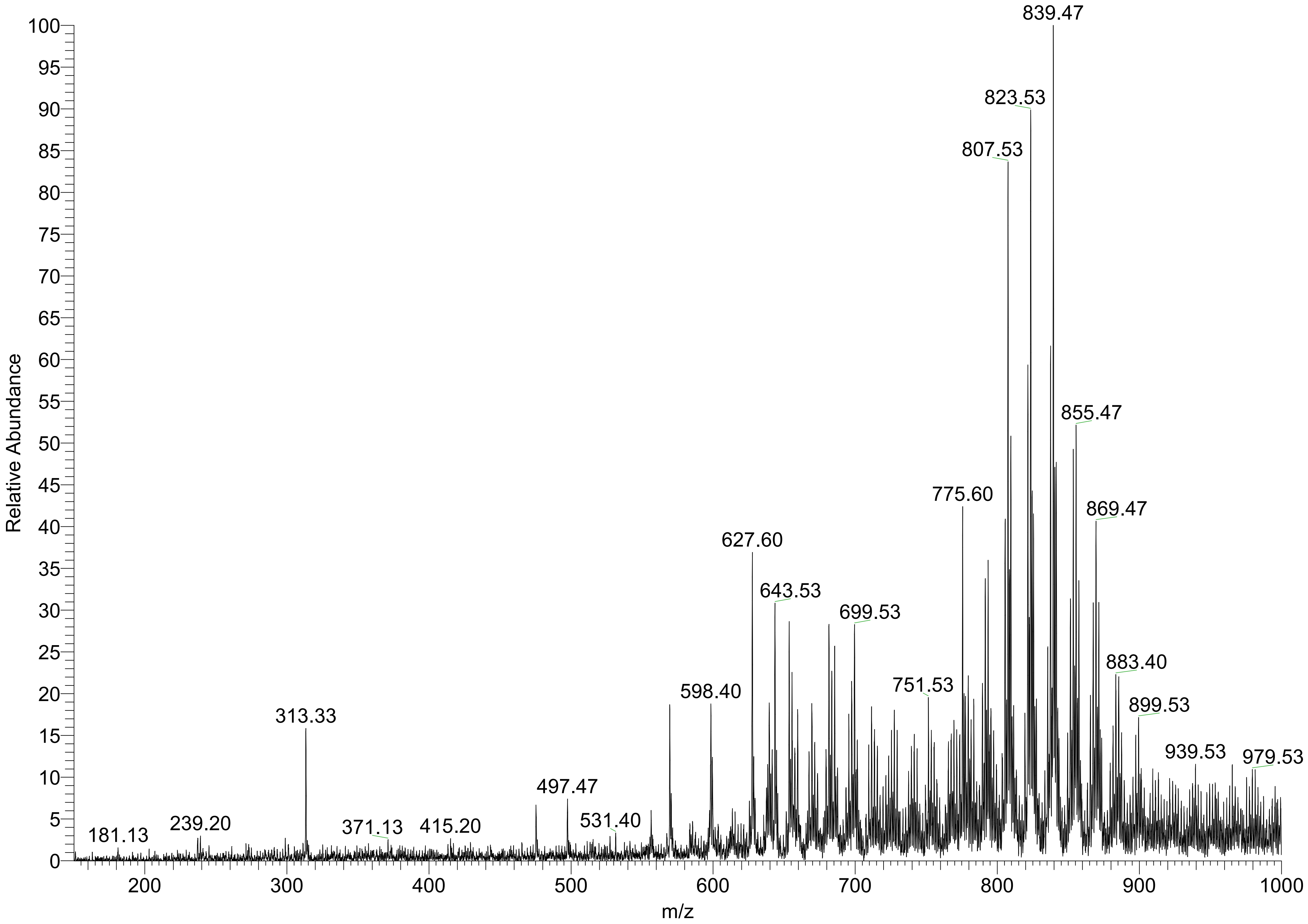

E:\dan_evans\RAW FILES\100%\S9 08/16/18 15:25:28 S9

S9 #1-50 RT: 0.01-0.75 AV: 50 NL: 7.68E5
T: + p ESI ms [150.00-1000.00]

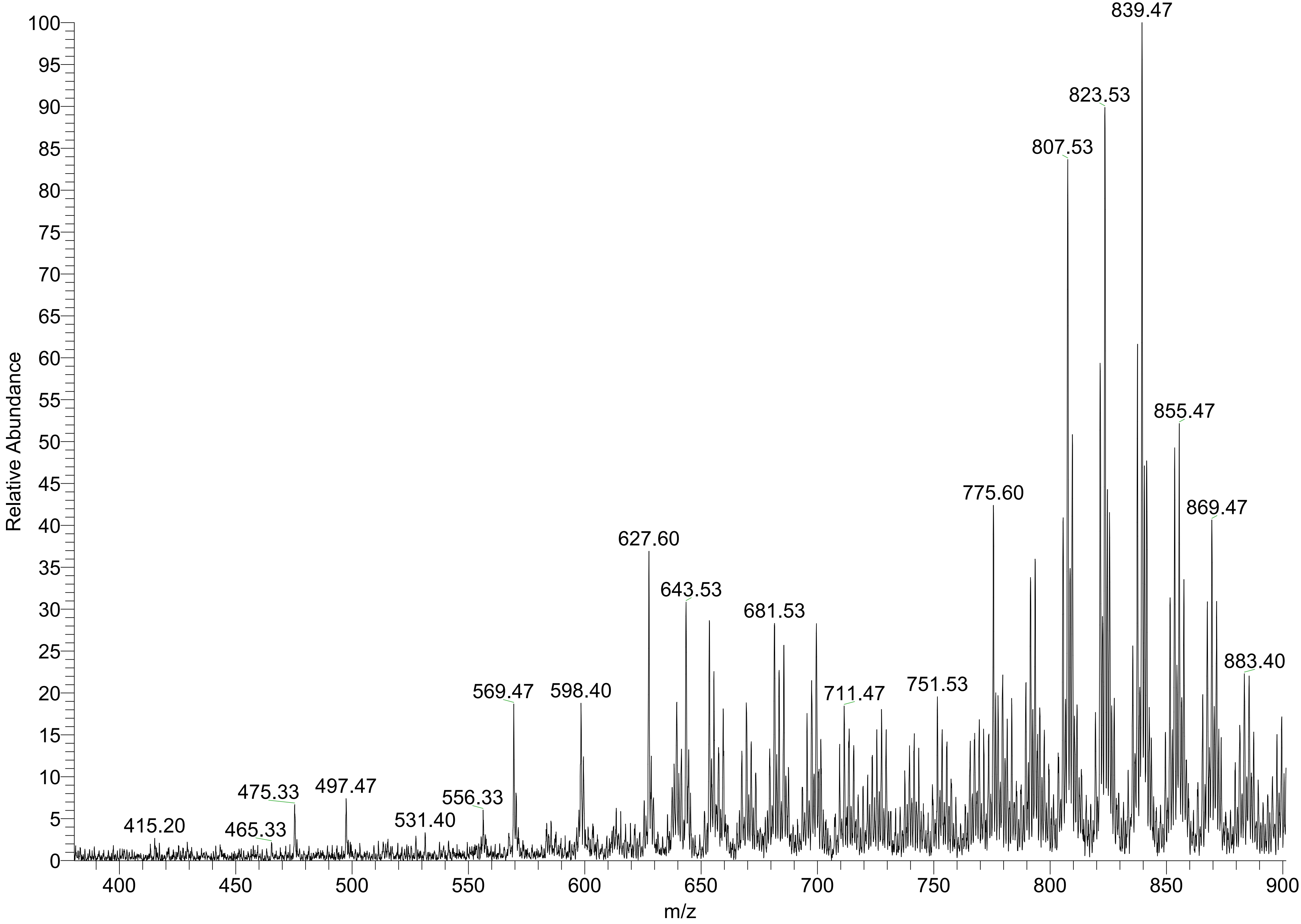

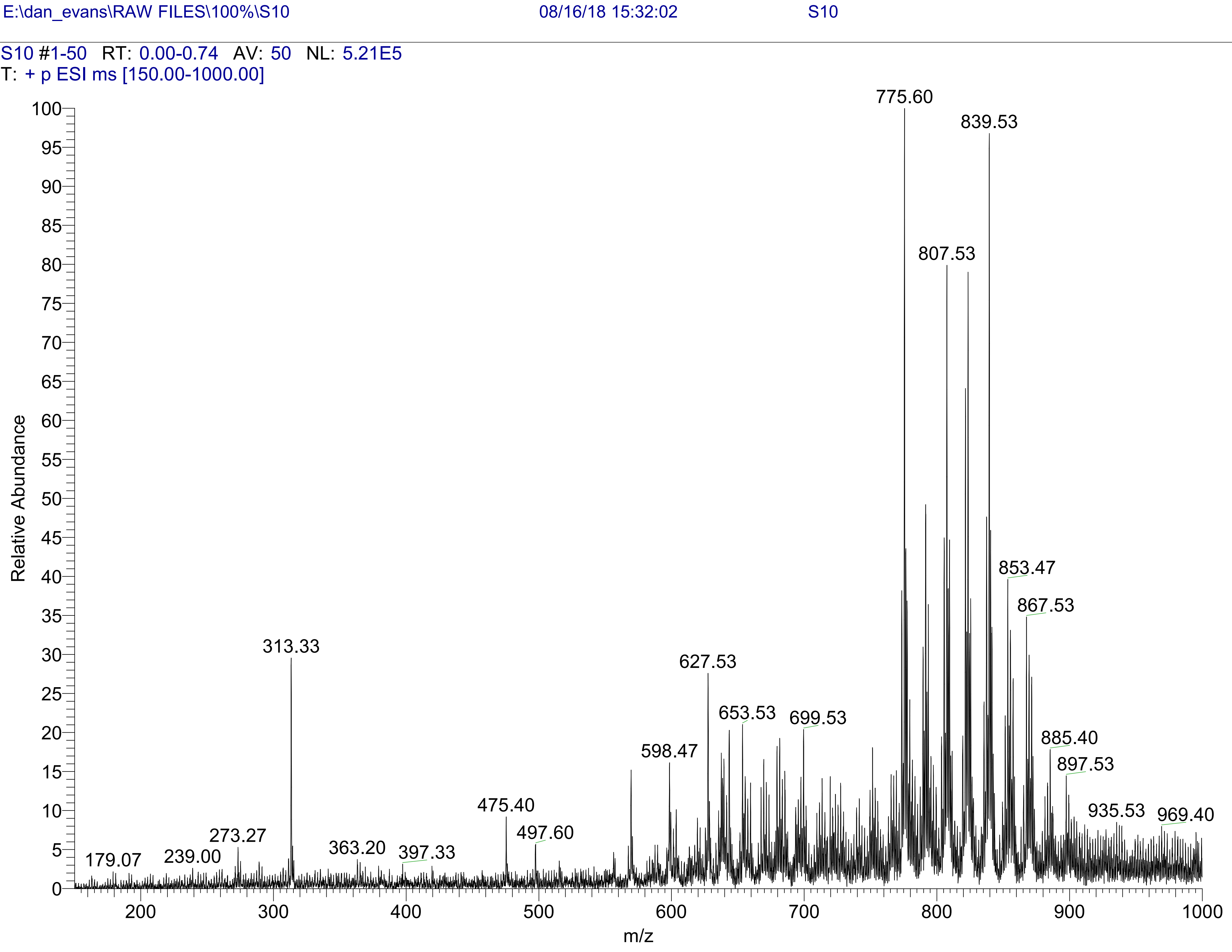
E:\dan_evans\RAW FILES\100%\S10
08/16/18 15:32:02
S10
S10 #1-50 RT: 0.00-0.74 AV: 50 NL: 5.21E5
T: + p ESI ms [150.00-1000.00]
775.60
839.53
807.53
853.47
867.53
313.33
627.53
653.53
699.53
885.40
598.47
897.53
475.40
935.53
969.40
497.60
273.27
363.20
397.33
179.07
239.00
Relative Abundance
m/z

E:\dan_evans\RAW FILES\100%\S10 08/16/18 15:32:02 S10

S10 #1-50 RT: 0.00-0.74 AV: 50 NL: 5.21E5
T: + p ESI ms [150.00-1000.00]

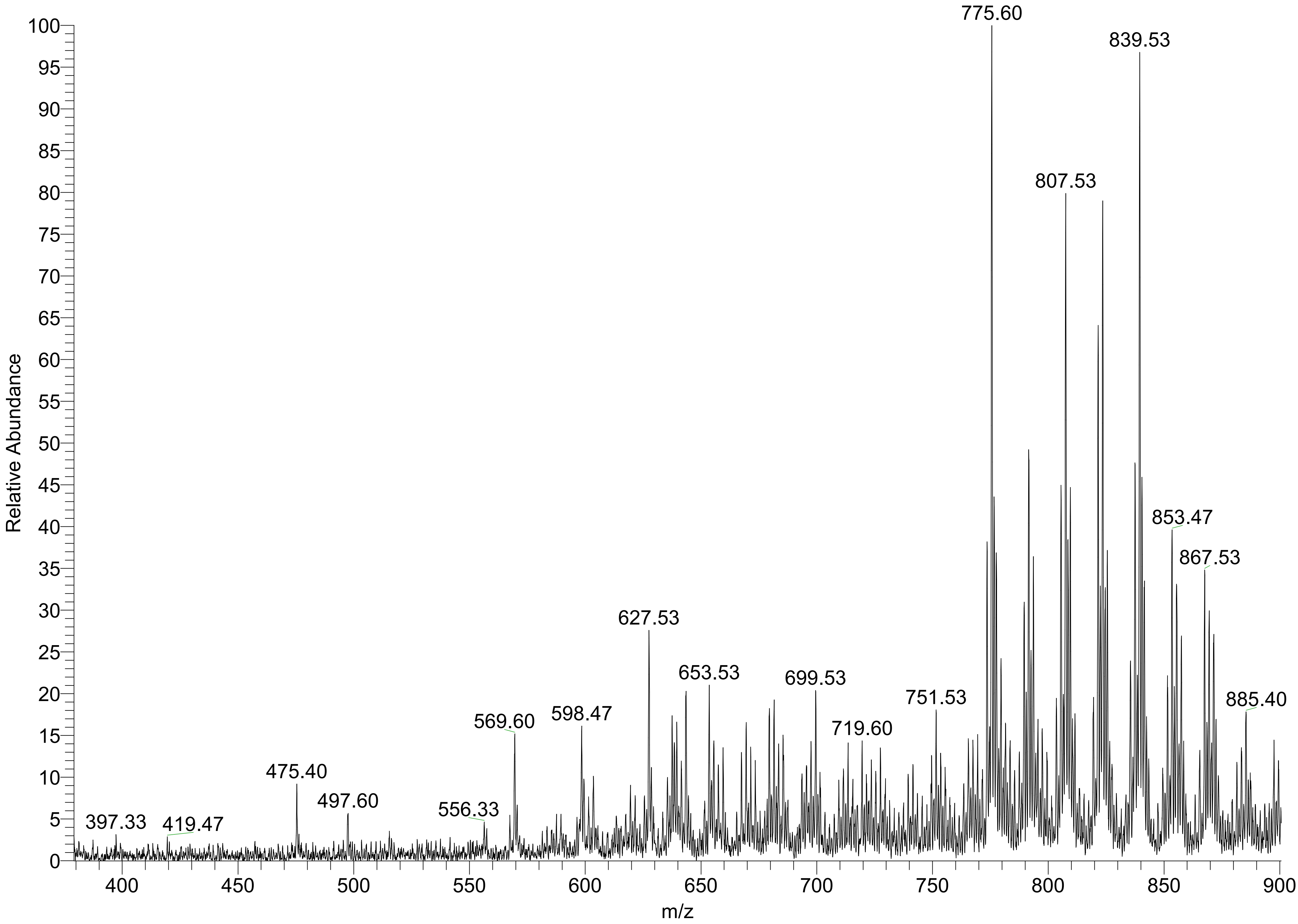

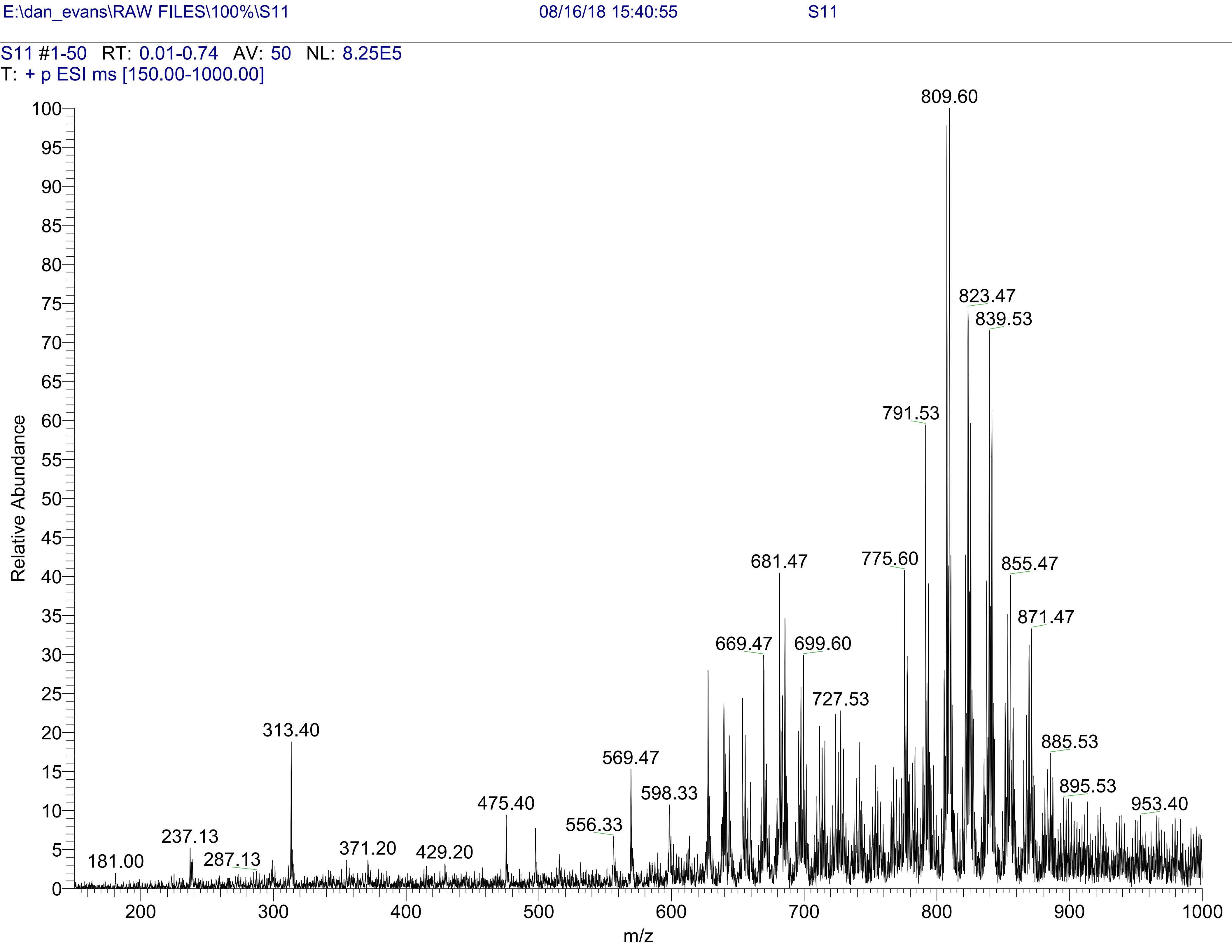

E:\dan_evans\RAW FILES\100%\S11
08/16/18 15:40:55
S11
S11 #1-50 RT: 0.01-0.74 AV: 50 NL: 8.25E5
T: + p ESI ms [150.00-1000.00]
Relative Abundance
m/z
809.60
823.47
839.53
791.53
775.60
681.47
855.47
871.47
669.47
699.60
727.53
885.53
895.53
953.40
313.40
569.47
598.33
475.40
556.33
237.13
287.13
371.20
429.20
181.00

E:\dan_evans\RAW FILES\100%\S11 08/16/18 15:40:55 S11

S11 #1-50 RT: 0.01-0.74 AV: 50 NL: 8.25E5
T: + p ESI ms [150.00-1000.00]

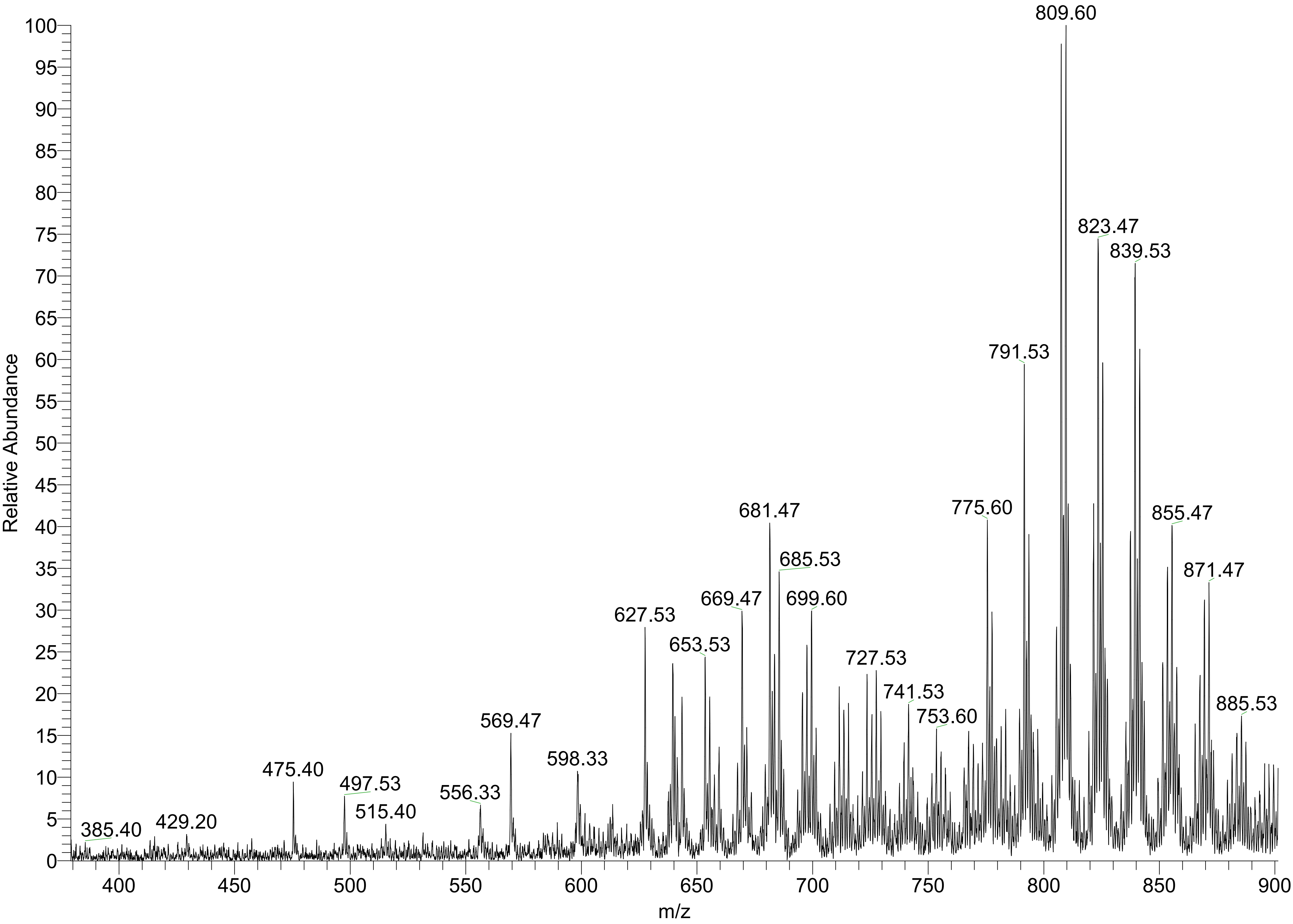

E:\dan_evans\RAW FILES\100%\S12 08/16/18 15:46:27 S12

S12 #1-50 RT: 0.00-0.74 AV: 50 NL: 6.82E5
T: + p ESI ms [150.00-1000.00]

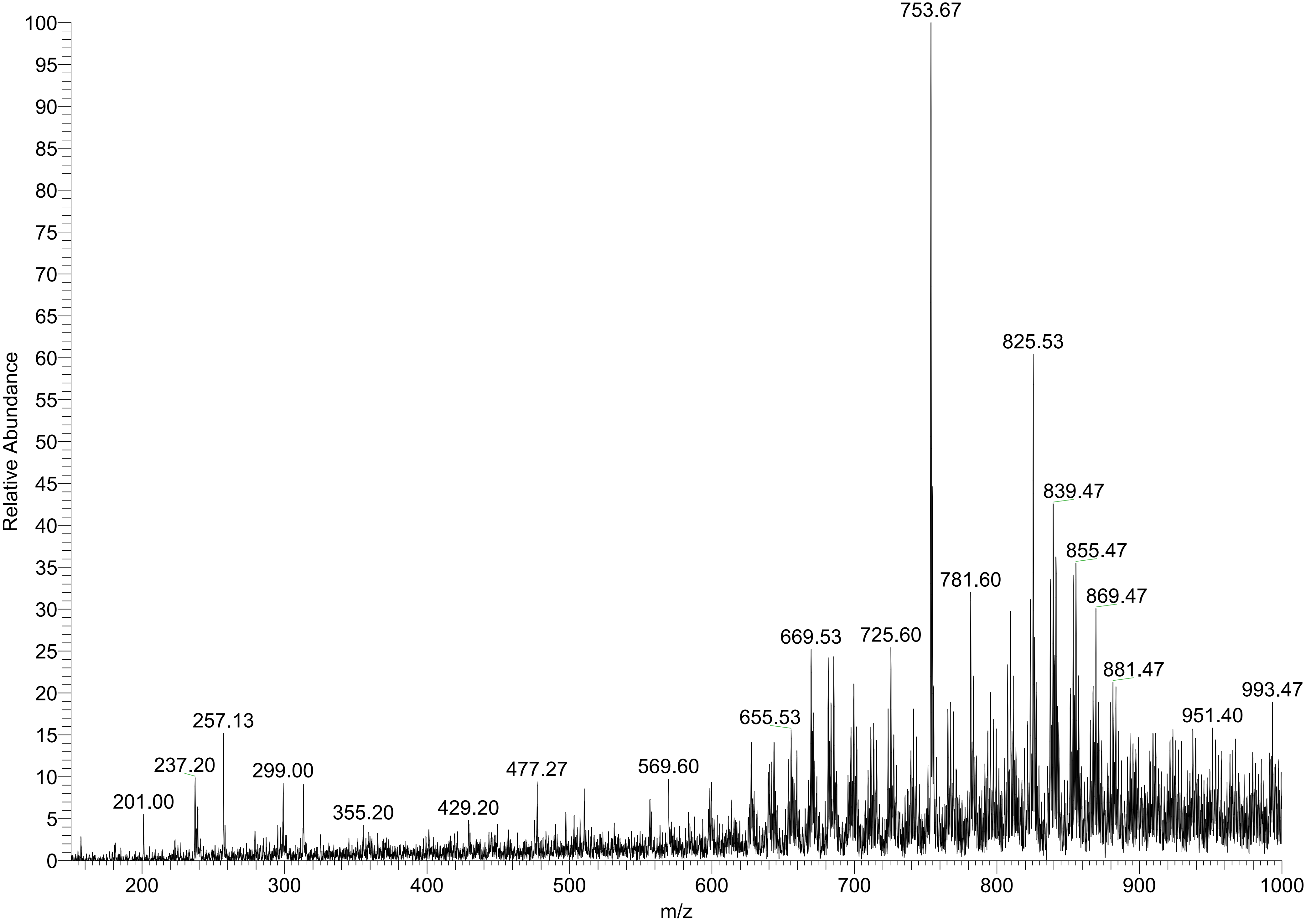

E:\dan_evans\RAW FILES\100%\S12 08/16/18 15:46:27 S12

S12 #1-50 RT: 0.00-0.74 AV: 50 NL: 6.82E5
T: + p ESI ms [150.00-1000.00]

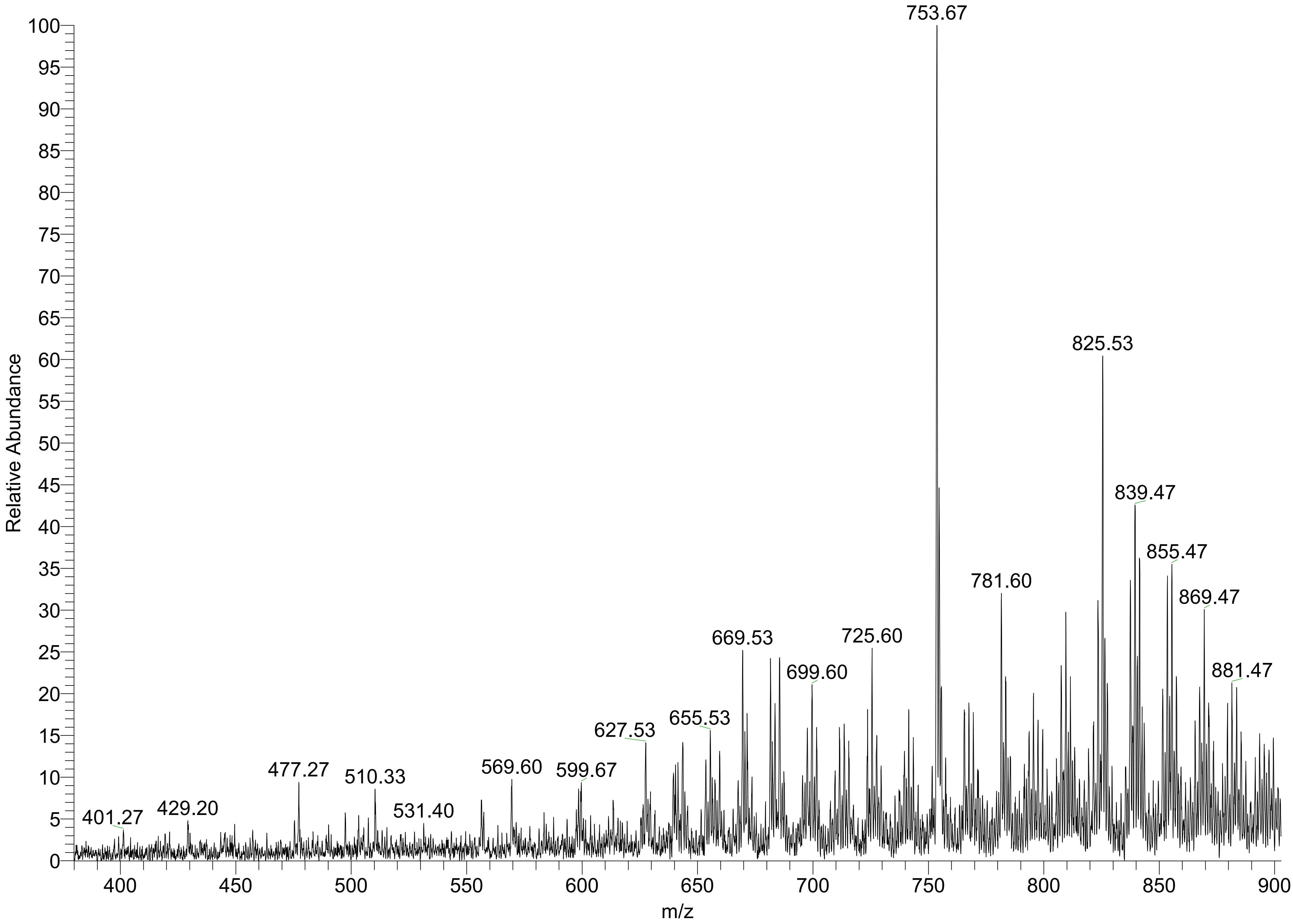

E:\dan_evans\RAW FILES\100%\S13 08/16/18 15:51:07 S13

S13 #1-50 RT: 0.01-0.74 AV: 50 NL: 4.11E5
T: + p ESI ms [150.00-1000.00]

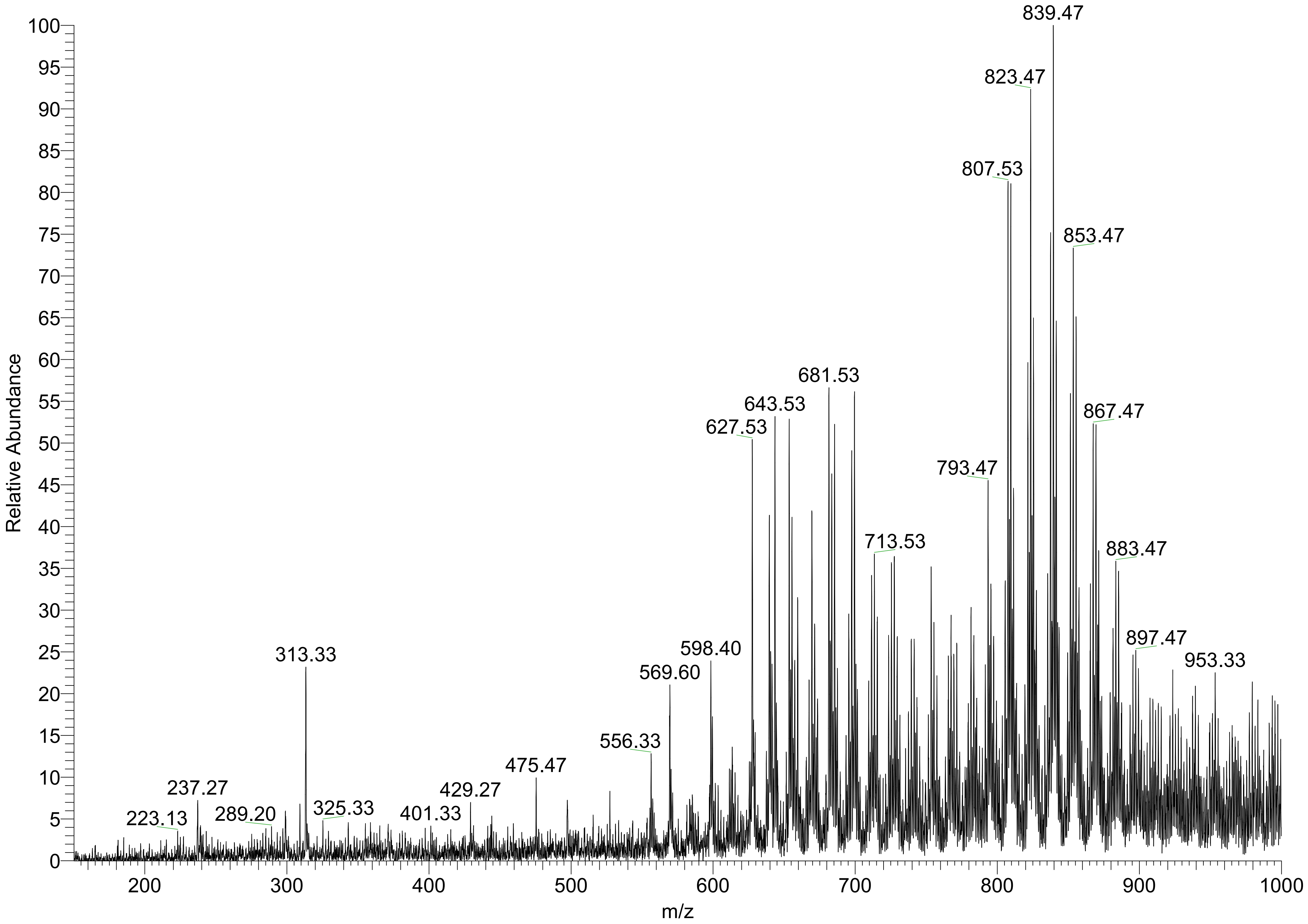

S13 #1-50 RT: 0.01-0.74 AV: 50 NL: 4.11E5
T: + p ESI ms [150.00-1000.00]

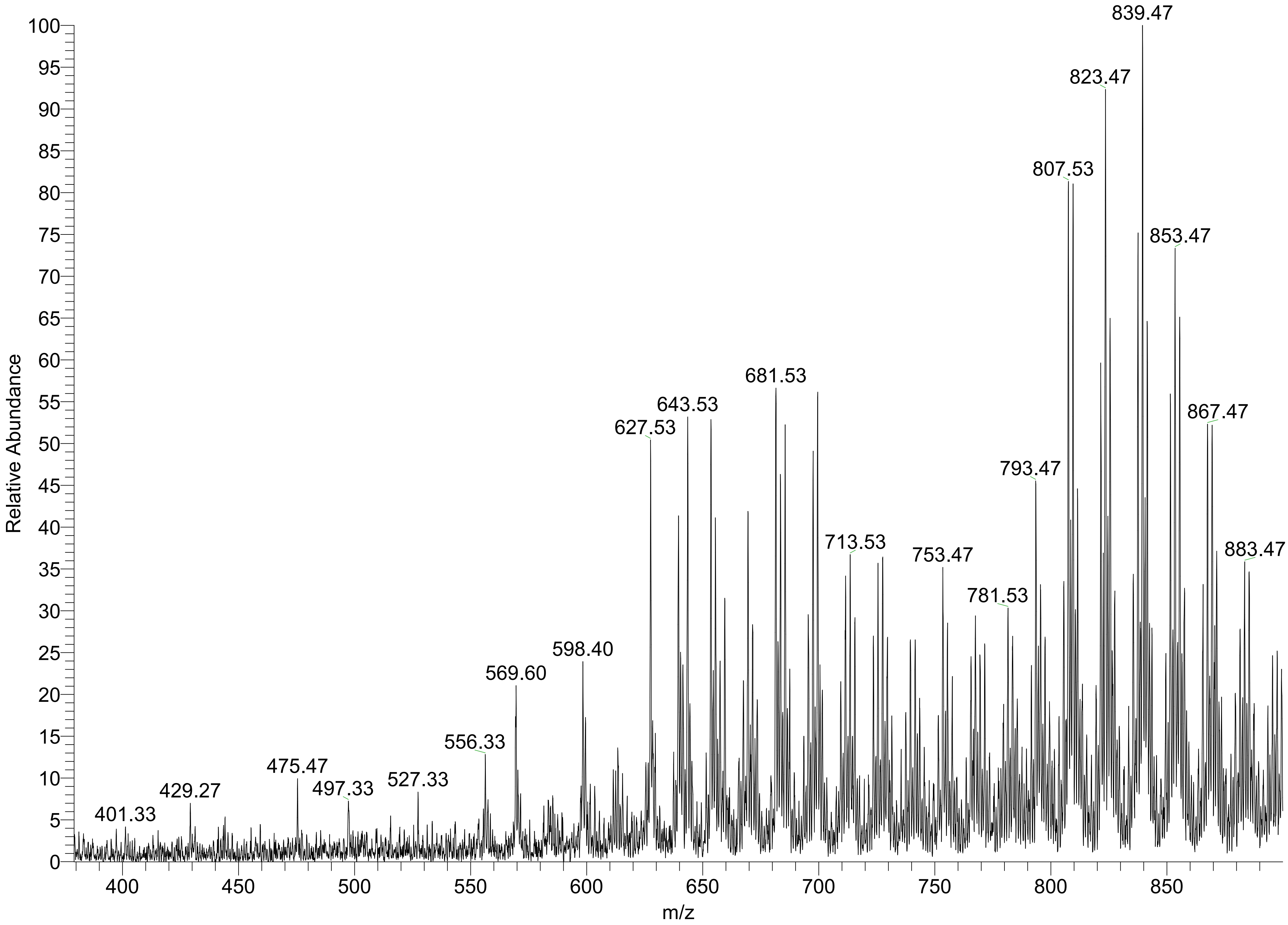

Supplement: Supplementary file 1 [file toxins-12-00111-s001.zip › toxins-705066 SUPP.pdf]
